# Possible roles of mechanical cell elimination intrinsic to growing tissues from the perspective of tissue growth efficiency and homeostasis

**DOI:** 10.1371/journal.pcbi.1005651

**Published:** 2017-07-13

**Authors:** Sang-Woo Lee, Yoshihiro Morishita

**Affiliations:** Laboratory for Developmental Morphogeometry, RIKEN Quantitative Biology Center, Kobe, Japan; Oxford, UNITED KINGDOM

## Abstract

Cell competition is a phenomenon originally described as the competition between cell populations with different genetic backgrounds; losing cells with lower fitness are eliminated. With the progress in identification of related molecules, some reports described the relevance of cell mechanics during elimination. Furthermore, recent live imaging studies have shown that even in tissues composed of genetically identical cells, a non-negligible number of cells are eliminated during growth. Thus, mechanical cell elimination (MCE) as a consequence of mechanical cellular interactions is an unavoidable event in growing tissues and a commonly observed phenomenon. Here, we studied MCE in a genetically-homogeneous tissue from the perspective of tissue growth efficiency and homeostasis. First, we propose two quantitative measures, cell and tissue fitness, to evaluate cellular competitiveness and tissue growth efficiency, respectively. By mechanical tissue simulation in a pure population where all cells have the same mechanical traits, we clarified the dependence of cell elimination rate or cell fitness on different mechanical/growth parameters. In particular, we found that geometrical (specifically, cell size) and mechanical (stress magnitude) heterogeneities are common determinants of the elimination rate. Based on these results, we propose possible mechanical feedback mechanisms that could improve tissue growth efficiency and density/stress homeostasis. Moreover, when cells with different mechanical traits are mixed (e.g., in the presence of phenotypic variation), we show that MCE could drive a drastic shift in cell trait distribution, thereby improving tissue growth efficiency through the selection of cellular traits, i.e. intra-tissue “evolution”. Along with the improvement of growth efficiency, cell density, stress state, and phenotype (mechanical traits) were also shown to be homogenized through growth. More theoretically, we propose a mathematical model that approximates cell competition dynamics, by which the time evolution of tissue fitness and cellular trait distribution can be predicted without directly simulating a cell-based mechanical model.

## Author summary

When genetically different cell populations are mixed, there is competition between cells such that losing cells are eliminated from a tissue. Such cell elimination is also observed during normal development in genetically-homogeneous tissues. In addition to the identification of key genes and molecular mechanisms related to these phenomena, the relevance of cell/tissue mechanics has been reported as a possible common mechanism of elimination. Here, we examined these mechanisms and possible functions of mechanical cell elimination (MCE) from the perspective of tissue growth efficiency and homeostasis. Using mechanical simulations of tissue growth processes, we identified key parameters of cellular mechanical/growth properties that determine elimination rates or cellular fitness (defined as the difference between cell division and elimination rate). Based on these results, we propose mechanical feedback mechanisms that could improve tissue growth efficiency and density/stress homeostasis. Furthermore, when cells with different mechanical traits are mixed, we found that MCE could drive a drastic shift in cell trait distribution, thereby improving tissue growth efficiency through the selection of cellular traits. With this, cell density, stress state, and phenotype were also shown to become homogenous. Our results will permit the elucidation of the mechanisms of intrinsic tissue defense against abnormal cells by their elimination through mechanical cell-cell interactions.

## Introduction

In 1975, Morata and Ripoll analyzed the mosaic system of the *Drosophila* imaginal disc composed of wild type cells and mutant cells of ribosomal protein, and found that mutant cells underwent apoptosis and were eliminated from the tissue [1]. This was the first report of cell competition resulting from local cell-cell interaction. Subsequent work has shown that the competition phenomenon is widely present, not only in insects but also in vertebrates, and that the elimination of cells is realized through various processes such as cell death, phagocytosis, or live cell extrusion [2–4]. The process has close connections with important biological events such as tumor formation and tissue size regulation. Thus, it has attracted attention from a variety of fields [5,6]. As potential mechanisms of cell competition, related molecules and/or signaling pathways have been identified [7,8]. Moreover, recent reports have shown mechanical relevance as well as chemical or molecular mechanisms [7,9]; for example, Bielmeier et al. found that cells with mutations in genes that determine cell fate were extruded from a tissue by a common mechanical process [10]. In addition, de la Cova et al. reported that in the *Drosophila* imaginal disc, the effect of growth of clone did not reach beyond the AP compartment boundary [6], suggesting that cell elimination is influenced by mechanical constraints.

Interestingly, recent live imaging studies have shown that even when a population is genetically homogeneous, a non-negligible number of cells are extruded from developing tissues. For instance, it was reported that at pupal stages of *Drosophila* wing development, about 1000 cells are extruded when the number of cells constituting the wing tissue increases from 4000 to 8000, i.e. 20% of newly born cells are eliminated [11,12]. Similar live cell extrusion was observed around the midline of Notum closure [4,12]. In addition to epithelial development, in a culture system using MDCK cells, when cell density was artificially increased, some cells were excluded until the original density was restored [13]. In these cell elimination processes, there is no a priori program that selects which cells are lost, and these mechanisms were mostly explained in a mechanical context. On the other hand, Clavería et al. found that in early mouse development, competition is based on differences in the expression level of the Myc gene [14], identifying the existence of chemical signals which determine the relative merits between cells.

**Fig 1:**
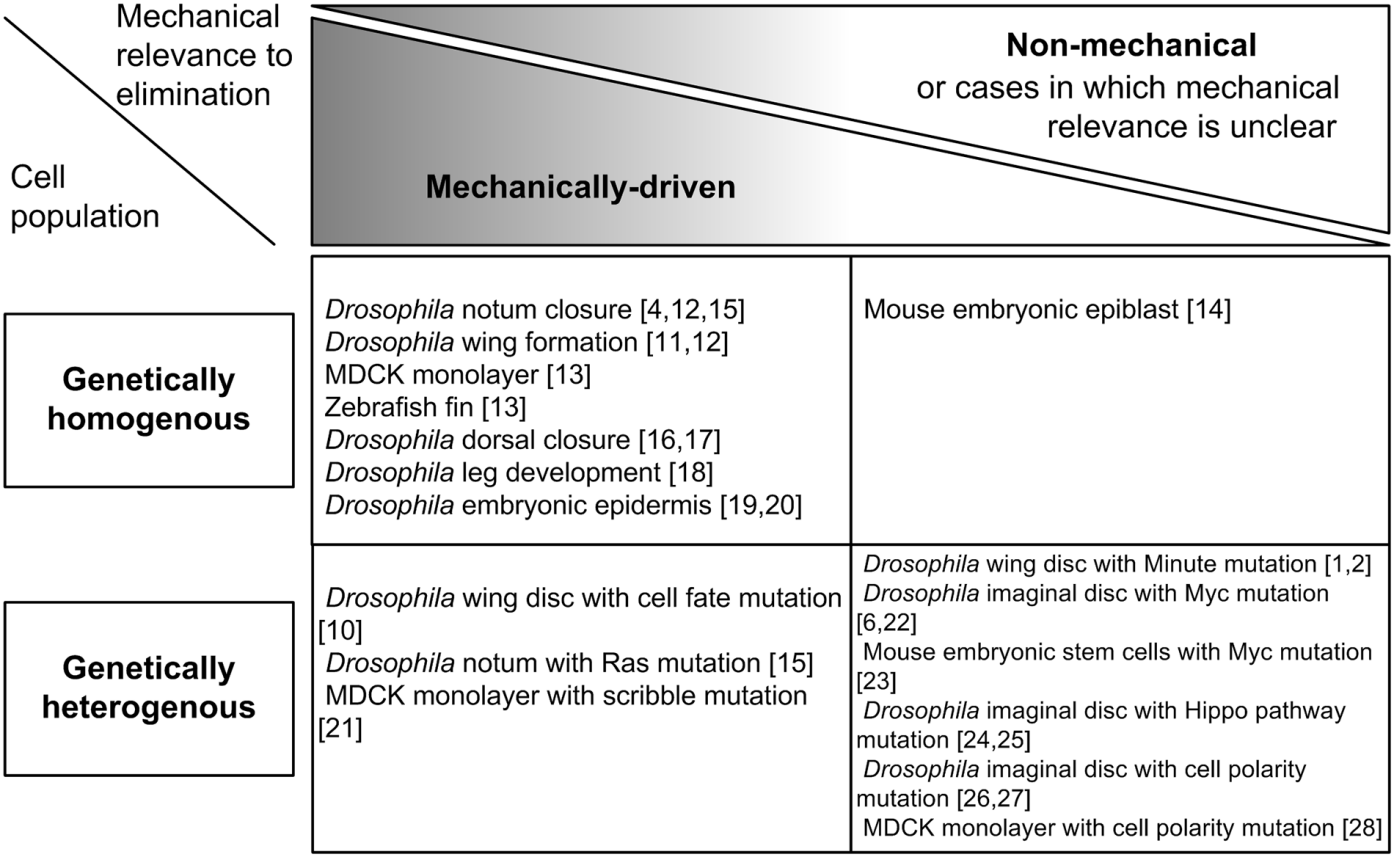
A classification of cell competition phenomena. We classified the experimental observations of cell competition based on two criteria. One is whether the elimination of cells is based on genetic differences or not; i.e. the focal cell population is either genetically homogeneous or heterogeneous. The other is the mechanical relevance; i.e. cell elimination is a mechanically-driven or non-mechanical event. As described in the text, the latter criterion is not completely exclusive and thus we included cases in which the mechanical relevance is unclear into the “non-mechanical” category. In this study we mainly focus on the case in the upper-left, i.e. mechanical cell elimination from a genetically-homogeneous growing tissue.

In this manner, the elimination of a portion of cells from a tissue or competition between cells is not necessarily due to a difference in genetic background. By regarding cell elimination from genetically-homogeneous cell populations as a form of broad-sense cellular competition, the experimental observations described above can be classified by the following criteria (Fig 1). The first criterion is whether the elimination is based on genetic differences or not, i.e. the focal cell population is “genetically-homogeneous” or “genetically-heterogeneous”. These are completely exclusive. Next, as a mechanism of elimination, the cases can be classified based on their mechanical relevance. Of course, this classification is not completely exclusive; for instance, cell-cell mechanical interactions might trigger the upregulation of previously identified cell death signaling pathways, while in another case, cell-cell chemical interactions through membrane-bound and/or secreted molecules might induce changes in mechanical cellular properties that enable the easy extrusion of cells from a tissue. Here, we used two kinds of tags, “mechanically-driven” and “non-mechanical” (or cases in which the mechanical relevance is unclear; Fig 1). [1,2,4,6,10–28]

In this study, we mainly focus on the mechanisms of mechanical cell elimination (MCE) from a genetically-homogeneous growing tissue for the following reasons. First, this issue is related to tissue growth efficiency, in other words, “how often do cells newly-born by proliferation contribute to tissue growth” and “how can tissues grow efficiently with less energy waste due to elimination?” Understanding the relationship between MCE and growth efficiency is important because tissues or individuals with higher growth efficiency have evolutionary advantages due to their faster growth (i.e., shorter time to reach their target sizes) and/or higher survival probability under environments with limited energy resources. Furthermore, as we will discuss later, cellular mechanical/growth parameters (also called cellular traits) generally show a distribution even if the genetic background of the cells is the same. Such phenotypic variation could induce cell-cell competition and change the distribution of cellular traits with an increase in cell population size. Since MCE in the presence of phenotypic variation can be regarded as a specific case of cell competition between populations of cells with different genetic backgrounds, examining what happens during the growth of such a mixed population of cells with different parameters will enhance our understanding of the mechanisms of cell competition (especially in relation to mechanical aspects). Secondly, it is likely that MCE is also related to tissue homeostasis. As shown in the density recovery experiment described above [4,13], if MCE is a kind of mechanical response to perturbations by extrinsic forces or cell division, it could function to maintain uniform cell density and/or stress distribution in a growing tissue. As one possible mechanism to maintain (local) cell density, the density or stress-dependent regulation of cell proliferation was proposed [29–32]. MCE could be another possible mechanism for cell density homeostasis. In addition, in the presence of phenotypic variation, competition could lead to the homogenization of cellular phenotypes. Lastly, from a more theoretical perspective, by focusing on the mechanical aspects, we can approach cell-elimination/competition independent of specific chemical signaling and gene regulation, the details of which are not well understood. Since the mechanical and growth parameters of cells are basically identical in a population, the rate of elimination due to mechanical cell-cell interactions can be uniquely determined for each population with a given set of mechanical parameters. The difference between the proliferation rate and elimination rate provides a net growth rate from which the fitness of the population can be quantified.

With this motivation, our aims are as follows. The first one is to clarify the dependence of mechanical/growth parameters on the cell elimination rate or net growth rate (fitness) and to find common geometrical/mechanical determinants of the elimination-rate/fitness. To achieve these aims, we introduce two quantities to define and measure fitness at the cellular and tissue levels. Regarding each cell population with a certain growth/mechanical trait as a “species”, the time derivative of its logarithmic growth curve defines the cellular fitness of the species. On the other hand, the tissue level fitness is defined as the average of cellular fitness between species weighted by the frequency of each species. When the traits of all cells in a tissue are identical (i.e., a pure population), the cellular and tissue level fitness are equivalent. In the presence of phenotypic variation, both cellular and tissue level fitness vary over time as a result of changes in the frequency distribution of cell types constituting the focal tissue through competition between them. After defining the fitness, by performing numerical simulations with a vertex dynamics model which is used in many studies of the mechanics of epithelial tissues [31,33–36], we examine the dependence of cell elimination rate or cellular fitness on mechanical/growth parameters of the cells. In contrast to experimental studies, we can independently control each parameter in the model, which is the biggest advantage of simulation-based studies. We show that the dependence could be summarized by how those parameters affected geometrical and stress heterogeneities within tissues, and that MCE functions to homogenize cell density and stress state within a tissue. Based on the simulation results, the second aim is to propose possible feedback mechanisms in which mechanical parameters of each cell are regulated depending on its stress state to improve tissue growth efficiency and homeostasis. These mechanisms could reduce the energy loss resulting from cell elimination, and homogenize cell density or tissue stress. Since the energy required for growth is proportional to the number of cells produced, the difference in the elimination rate becomes a greater advantage as tissue size increases. Interestingly, under the proposed feedback regulation, the geometrical and stress heterogeneities between cells were incompatible. By controlling geometrical heterogeneity, the elimination rate could be reduced but tissue stress heterogeneity increased (i.e. density homeostasis is achieved but stress homeostasis is impaired). In contrast, controlling the stress heterogeneity increased both geometrical heterogeneity and the elimination rate (i.e. stress homeostasis is achieved but density homeostasis is impaired). Finally, we examine what happens through competition in a population where cells with different mechanical traits are mixed. When daughter cells epigenetically inherit their parental traits (the degree of inheritance was quantified as heritability), the trait distribution within the tissue drastically changes with tissue growth, resulting in an increase in fitness at the tissue level. This clearly demonstrates that cell competition through MCE can improve tissue growth efficiency through the selection of mechanical cell traits, i.e. intra-tissue “evolution”. Furthermore, through selection, cell density, stress within a tissue, and cellular phenotype are homogenized, which is another possible role for competition through MCE. From a more theoretical perspective, we propose another differential equation model for competition dynamics that permits us to a calculate the approximate time evolution of tissue-level fitness and trait distribution. The model is useful for predicting the outcome when tissue size grows much larger, e.g., reaches a fully-developed size with ~10^6^−10^7^ cells, because direct simulations of a cell-based mechanical model require an immense amount of computation time.

## Results

### Introducing fitness measures at the cellular and tissue levels

Previous studies have introduced the concept of fitness to discuss the intensity of cell competition [8,37,38]. The main focus was relative survivability, that is, which population survives when different populations with different genetic backgrounds are mixed. In this study, we aim to reveal quantitative effects of cell competition through MCE on tissue growth dynamics. To do so, we now propose another quantitative measure of fitness, which differs from relative survivability.

In experimental observations and in tissue growth simulations, the growth curve of a developing tissue, i.e. temporal changes in the total number of cells within a growing tissue, *g*(*t*), can be locally approximated by an exponential function (during a certain period) [11,39,40]. Thus, its exponent, representing net growth rate or growth efficiency, defines the fitness of a focal tissue at each time *t*:

$$\phi_{Tissue}(t)=\frac{d}{dt}\text{log}g(t).$$ (1)

Assuming that growth dynamics is modeled by *dg*(*t*)/*dt* = (*μ*(*t*)-*m*(*t*))*g*(*t*), the fitness becomes simply *μ*(*t*)-*m*(*t*), where *μ*(*t*) is the tissue growth rate through cell proliferation. As stated before, in actuality, not all cells produced by division survive and contribute to the increase in tissue size. Some cells are lost from the tissue by extrusion or apoptosis, the rate of which (termed mortality) is represented by *m*(*t*). In this study, the cell elimination rate is regarded as a key factor in determining cell mortality within growing tissues. Rewriting the fitness as *μ*(*t*)(1-*m*(*t*)/*μ*(*t*)), the term (1-*m*(*t*)/*μ*(*t*)) or *m*(*t*)/*μ*(*t*) indicates the energy efficiency (the contribution ratio of produced cells to tissue growth) or the loss of energy (the waste of healthy cells). In this way, tissue fitness is determined by both the growth rate and energy efficiency at each time.

As an ideal situation, when a focal tissue is composed of cells with exactly the same traits in mechanical and/or growth properties (called a pure population), the fitness at the tissue level can also be regarded as the fitness of a cell with that focal trait, *ϕ*_*Cell*_ = *ϕ*_*Tissue*_. In this case, as shown later, the value of fitness is almost constant (when the growth rate is sufficiently slow). Using cell cycle *T*, the tissue growth rate *μ* is given by (log 2)/*T*. Regarding the mortality *m*, when defining the cell elimination rate as the ratio of the number of eliminated cells to that of newly-born cells by cell division per unit increment of tissue size: *ε* = *N*_*eliminated*_/*N*_*produced*_, the relationship *m* = -(log(1-*ε*/2))/*T* holds (see the Models section for details). In the next three sections, we consider such a pure cell population to examine how and by what mechanisms cell mechanical/growth properties affect fitness, especially mortality through cell elimination.

Consider next a tissue that is composed of cells with different traits (called a mixed population). Here, the net growth rate at the tissue level and that at the cellular level for each population with each trait are different in general. Denoting the cellular fitness with *i*-th trait by *ϕ*_*Cell*,*i*_, the tissue fitness is given by:

$$\phi_{Tissue}(t)=\sum_{i}f_{i}(t)\phi_{Cell,i}(t),$$ (2)

$$\phi_{Cell,i}(t)=\frac{d}{dt}\text{log}g_{i}(t),$$ (3)

where *f*_*i*_ is the frequency of cells with *i*-th trait in the entire tissue and *g*_*i*_(*t*) is the growth curve of the population with *i*-th trait. Importantly, in a mixed population, cellular fitness for each trait is generally not constant but varies with time. This is because the elimination rate of cells with a certain trait depends on the traits of its surrounding cells that mechanically interact with one another, and because the distribution of cellular traits throughout the entire tissue changes with time through selection based on the differences in cellular fitness.

Focusing on developmental advantages, fitness at the tissue level is more important than the cellular fitness. In the remaining subsections, we show how mechanical feedback and competition between different traits improve tissue fitness or growth efficiency.

### Dependence of the cell elimination rate on mechanical tissue properties and growth rules

**Fig 2:**
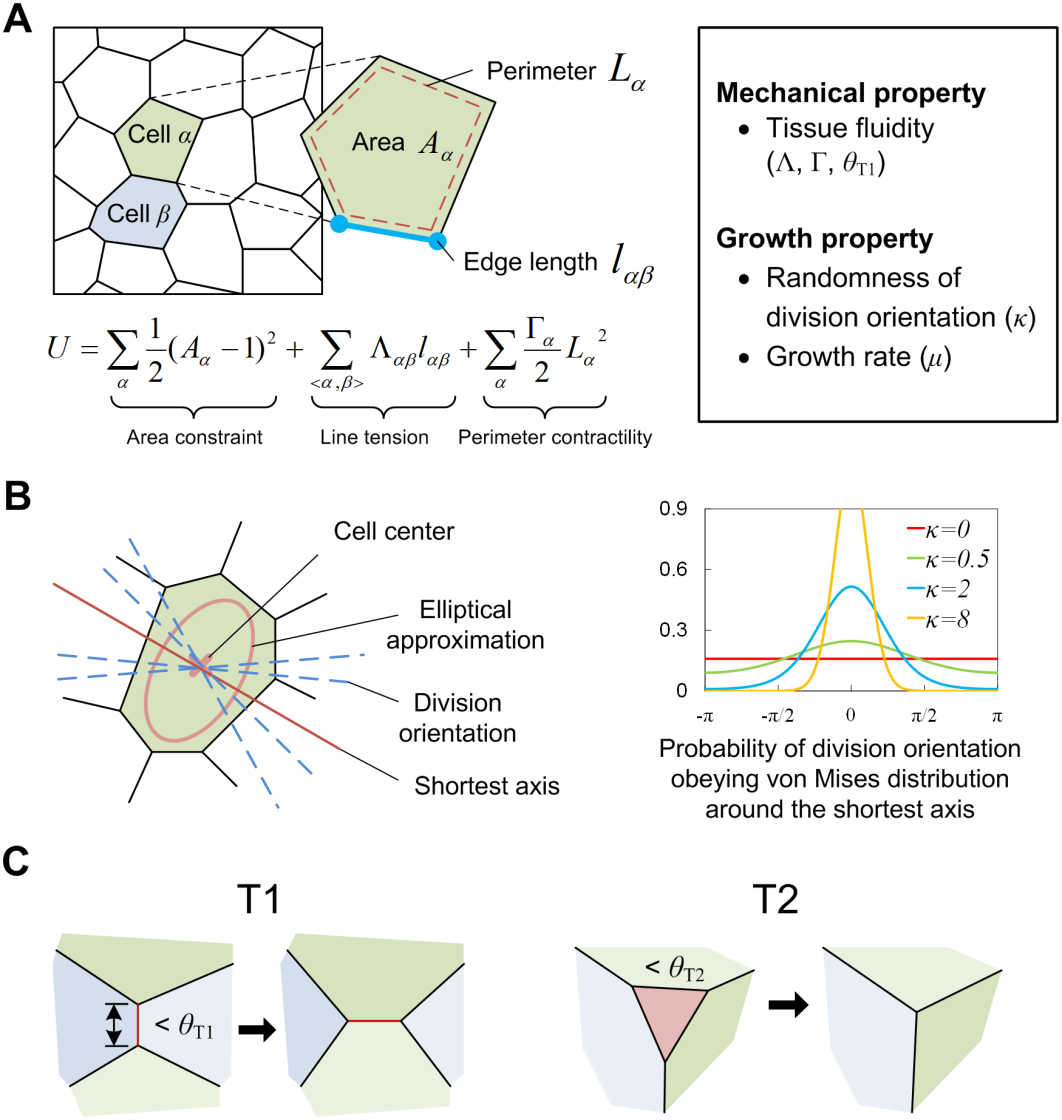
Settings for mechanical tissue simulation using a vertex dynamics model. (A) A schematic diagram of the vertex dynamics model. In this model, each cell is represented as a polygon formed by linking several vertices. Each vertex moves in a manner that decreases the energy *U* of the system. *U* is composed of three terms: area constraint, line tension and perimeter contractility. The mechanical traits of each cell are represented by two kinds of parameters Λ_*αβ*_ and Γ_*α*_. Λ_*αβ*_ is the coefficient for tension acting on a cell edge between cell *α* and cell *β* (blue line). The other parameter Γ_*α*_ is the contractility of the apical surface (red dashed line). (B) The rule of cell division orientation. A cell divides with an axis through its center (left). The orientation of the axis is a random variable obeying the von Mises distribution *f*(*θ*;*κ*) around the shortest axis *θ* obtained by elliptical approximation of the cell. The randomness can be regulated by a single parameter *κ* (right). (C) Cell rearrangement (T1-process) and elimination (T2-process). As a consequence of push-pull dynamics between cells in a growing tissue, the spatial rearrangement and elimination of cells occur. The rearrangement occurs when the edge length is less than the T1-threshold *θ*_T1_ (left). Elimination is implemented simply by removing the cell whose area is less than the T2-threshold *θ*_T2_, which is called a T2-process (right).

We started by systematically examining how the frequency of cell elimination depends on mechanical properties and the growth rules of epithelial cells using the vertex dynamics model (Fig 2A, also see the Models section). Specifically, we focused on tissue fluidity, cell division orientation, and proliferation rate. In the vertex dynamics model, each cell shape is represented as a polygon formed by linking several vertices, and each vertex moves so as to decrease energy function *U* of the system (see the Models section). *U* includes two parameters Λ and Γ (see the Models section for details). Λ is the coefficient for tension acting on a cell’s edge; stronger cell-cell adhesion and/or weaker contractility of actomyosin fibers along the edge correspond to a smaller value of Λ. The other parameter Γ is the coefficient for perimeter elasticity, the value of which is determined by the contractility of the actomyosin network over the apical surface of a cell [35]. Each cell has a clock representing the cell cycle. When the clock within a cell reaches a specific value *T*, the cell divides with an axis through its center and the clock is reset (note that the cell cycle includes slight stochasticity to avoid the synchronization of divisions; see the Models section for details). Regarding the division orientation, we modeled it as a random variable distributed around the shortest axis. By changing a single parameter in the distribution, the randomness of division orientation can be controlled (Fig 2B; also see the Models section). As a consequence of push-pull dynamics between cells through their divisions, a cell whose area is below a certain threshold (*θ*_T2_ = 0.2) will be removed (called T2 process; Fig 2C; also see the Models section). As mentioned in the introduction, there have been some reports on MCE. However, currently there is little known about whether a threshold in cell area for MCE exists and what the threshold value is. The only exception is a study by Marinari et al. in which they showed that in the case of *Drosophila* notum development, cells whose area was less than ~25% of the initial area were eliminated [4]. According to this report, we set *θ*_T2_ = 0.2. To confirm the generality of our results, we also examined cases with different values for *θ*_T2_ (specifically, 0.05, 0.1, and 0.3), and our results (shown below) did not change qualitatively. In addition, as clarified in the later subsection, cell size is highly correlated with stress state, and thus our assumption on the criterion for MCE, the existence of a cell size threshold for MCE, also includes another criterion, the existence of a threshold for stress acting on a cell.

Tissue fluidity, i.e. the liquid-like behavior of a tissue, increases for smaller values of Λ and/or Γ [35]. Intuitively, in those situations, each cell moves so as to maintain its apical area as near to the natural value (a given constant, see the Models section) as possible, leading to easier deformation and more frequent cell-cell rearrangements when forces due to tissue growth act on the cell. In contrast, for larger values of those parameters, the force for isotropic shrinking increases, resulting in less fluidity (see also the Models section). In the vertex dynamics model, cell rearrangement is implemented by the reconnection of vertex networks called a T1-process (Fig 2C), and thus the distance threshold for the reconnection (*θ*_T1_) also affects the tissue fluidity. Whether or not the value of *θ*_T1_ itself is a controllable parameter is unknown. However, observations have shown that in some situations cell intercalation frequently occurs, and in other situations it rarely occurs and multicellular rosette structures are formed instead [41], suggesting the existence of mechanisms that regulate the frequency of intercellular rearrangement.

**Fig 3:**
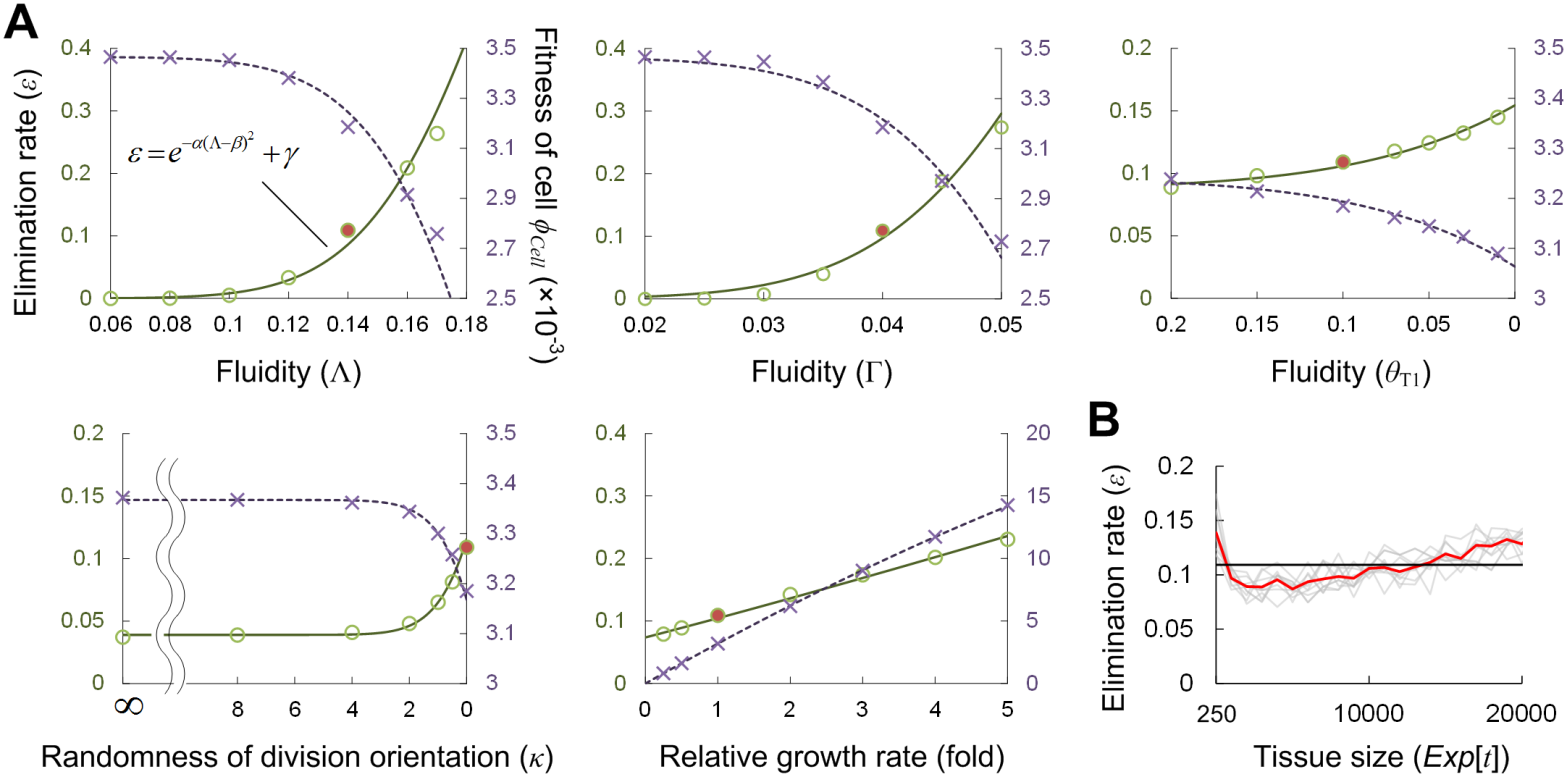
Cell elimination rate and fitness in a pure population. (A) Dependence of the cell elimination rate *ε* (green circles) and the cellular fitness *ϕ*_*Cell*_ (= *ϕ*_*Tissue*_) (purple crosses) on mechanical/growth parameters in simulations, which can be well approximated by Gaussian-type functions (solid green and dashed purple lines), *ε* = *Exp*[-*α*(*ζ*-*β*)^2^]+*γ*, where *ζ* represents any one of the mechanical/growth parameters (Λ, Γ, *θ*_T1_, *κ* and *μ*). Parameters: (*α*, *β*, *γ*) = (245, 0.24, 0) for Λ, (1800, 0.076, 0) for Γ, (12.5, 0.465, 0.0875) for *θ*_T1_, (0.095, 5.2, 0.039) for *κ*, (0.0016, 24.3, -0.31) for *μ*. (B) Time evolution of the elimination rate *ε* in a case with the reference parameter set (see the red circles in Fig 3A; Λ = 0.14, Γ = 0.04, *θ*_T1_ = 0.1, *κ* = 0 and *μ* = 3.47×10^−3^); the red line is the average at each time point over 10 trials shown by the gray lines. The black line is the temporal average of the red line. Since the variation in elimination rate is not large during tissue growth when the number of cells ranges from *N* = 250 to *N* = 20,000, we adopt its temporal average as the typical value of the elimination rate for each parameter set.

The upper panels in Fig 3A show the dependences of cell elimination rate *ε* and *ϕ*_*Cell*_ on the three parameters affecting the tissue fluidity. These three parameters have the same tendency, although the degree of dependence on the T1-threshold *θ*_T1_ is lower. With an increase in tissue fluidity, the elimination rate decreases or the fitness increases monotonically. In particular, the parameter dependence of *ε* can be approximated by a Gaussian-type function (Fig 3A), which is useful in calculating the time evolution of cellular/tissue fitness within a mixed cell population as will be shown later (see the final subsection). For a fixed parameter, the cell elimination rate *ε* and the cellular fitness *ϕ*_*Cell*_ are nearly constant during tissue growth as long as cell density is regarded as constant (Fig 3B).

As described above, the randomness of cell division orientation was introduced as a tissue growth rule (Fig 2B). It was controlled by a single parameter, the variance of division orientation around the shortest axis of each cell. Unexpectedly, the division orientation has a clear effect on the cell elimination rate. When a cell divides along the shortest axis, the elimination rate decreases compared to situations in which division is randomly oriented (Fig 3A). In regards to the growth rate, as expected, the elimination rate becomes higher as it increases (Fig 3A). In actuality, this tendency has been observed in a biological system. During development of the *Drosophila* notum, cell elimination occurred more frequently in the tissue of the mutant with the higher growth rate [4].

### Cell size variance, but not cell shape regularity or cell rearrangement frequency, is the common geometrical determinant of the elimination rate

As shown in the previous subsection, cell elimination naturally occurs as a consequence of tissue growth, and its rate depends on different mechanical/growth parameters. In order to find common factors for determining the elimination rates, we next searched for quantities whose values change with the same tendency as the elimination rate when mechanical/growth parameters change. Specifically, we focused on (i) cell shape regularity, which was quantified by elliptical approximation, (ii) the frequency of cell rearrangement (T1-process) and (iii) the variance in size between cells.

**Fig 4:**
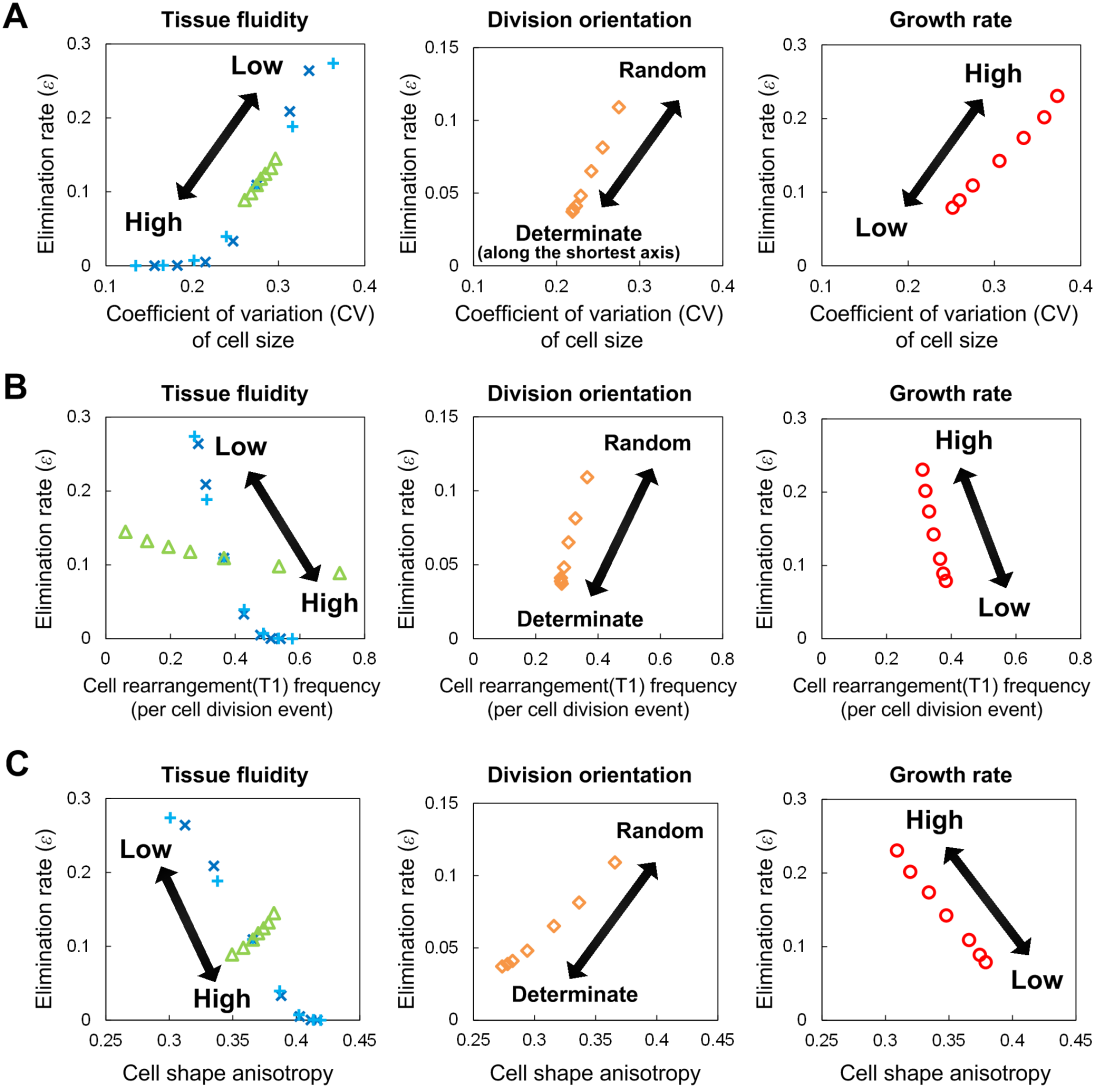
Cell size variance as the common geometrical determinant of the elimination rate. When the cellular mechanical/growth parameters change, geometrical quantities (specifically, variation in cell size, cell rearrangement frequency, and cell shape anisotropy) and elimination rate change. (A) For example, when the tissue fluidity (that is determined by Λ, Γ, and *θ*_T1_) increases (or decreases), both the variation in cell size and elimination rate decrease (or increase; top panel). Similarly, for changes in the other parameters, *κ* (which determines the randomness of division orientation) and *μ* (growth rate), both cell size variation and the elimination rate change with the same tendency. In this manner, the responses of cell size variation and elimination rate to changes in any parameter are highly and positively correlated. In contrast, the responses in cell rearrangement frequency (B) and cell shape anisotropy (C) to parameter change does not necessarily show the same tendency as the response in cell elimination; for instance, for changes in tissue fluidity and growth rate, the responses of rearrangement frequency and elimination rate are negatively correlated, whereas for changes in division orientation they are positively correlated. Thus, among these three geometrical quantities, only cell size variation had a response consistent with the response in elimination rate, and we can conclude that cell size variance is the common geometrical determinant of the cell elimination rate. Note that cell shape anisotropy was calculated as 1-(length of the shortest axis/length of the longest axis) after approximating each cell by an ellipse. All simulations were performed using a pure population. Symbols: blue crosses (Λ), cyan pluses (Γ), green triangles (*θ*_T1_), orange diamonds (*κ*), and red circles (*μ*).

**Fig 5:**
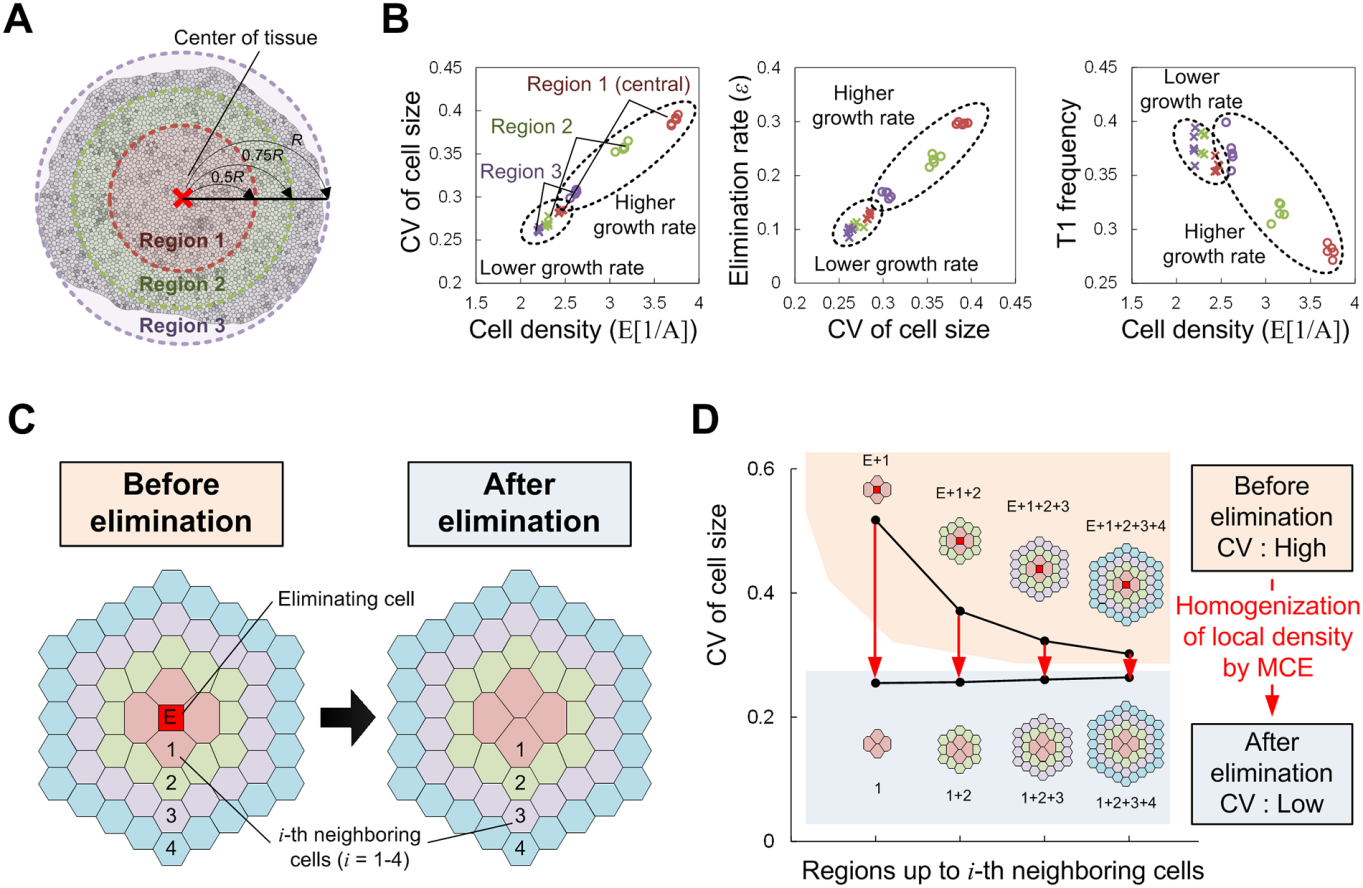
Relationship between spatial heterogeneity in cell density and MCE. (A) A schematic diagram of the calculation for regional dependence in cell density. The whole tissue was divided into three regions, and for each region the temporal averages in cell density, cell size variance, and cell elimination rate were calculated. (B) The relationships between the temporal averages in cell density and cell size variance (left), between the temporal averages in cell size variance and cell elimination rate (middle), and between the temporal averages in cell density and T1-frequency (right). When the growth rate is not high (e.g., for the reference value), all three values were nearly the same for all three regions. In contrast, in cases with a higher growth rate, those values were strongly dependent on region. More central regions have higher density, which inhibits smooth cell rearrangement (i.e. cell density and T1-frequency were negatively correlated) and thus leads to an increase in cell size variance and elimination. Cell density and size variance had a clear positive correlation, as did size variance and elimination rate. (C) A schematic diagram of the calculation for change in cell size variance around the elimination point before and after its occurrence. (D) Cell size variance clearly decreased due to MCE, demonstrating that recovery in the homogeneity of cell density (i.e., density homeostasis) is one possible role for MCE.

As shown in Fig 4A, only the variance in cell size has a high correlation with the elimination rate, indicating that cell size variance is the only geometrical determinant of the elimination rate. This is reasonable because cell elimination, i.e. the T2-process in the vertex dynamics model, is determined by cell size. However, it may be significant that the correlation with the remaining quantities (T1-frequency and cell shape regularity) is much lower. This can be interpreted as follows. Different mechanical and growth parameters affect the cell size variance (and thus cell elimination) in different ways (Fig 4). For example, for higher tissue fluidity (e.g., smaller Λ/Γ or larger *θ*_T1_), the increase in T1-frequency reduces the cell size variance, leading to a decrease in the elimination rate (left panels in Fig 4A and 4B). In contrast, biased division-orientation along the shortest axis reduces the elimination rate even though the frequency of the T1-process is much lower (middle panel in Fig 4B). In this case, the increase in cell shape regularity instead of T1-frequency likely reduces the cell size variance (middle panel in Fig 4C). In regard to growth rate, as shown in Fig 4B, a higher growth rate decreases the T1-frequency and relative tissue fluidity is lower, which could cause an increase in the cell size variance and elimination rate. A higher growth rate also increases the spatial heterogeneity of cell density (Fig 5B); the density becomes higher in more central regions of tissue as observed in the development of the *Drosophila* wing imaginal disc [42,43]. As shown in Fig 5B, cell density has a clear positive correlation with size variance. More central regions have higher density, which inhibits smooth cell rearrangement and thus leads to an increase in cell size variance and elimination (right panel in Fig 5B). Taken together, cell size variance is regulated in different ways and determines the cell elimination rate.

Since the inverse of the cell area corresponds to local cell density, the above result can also be interpreted as the spatial heterogeneity in cell density determines the cell elimination rate. As expected, the heterogeneity of cell density around an elimination point (defined as the CV of the inverse of cell size in the region including the elimination point) decreases after its occurrence, demonstrating that cell elimination can recover the homogeneity in cell density (Fig 5C and 5D).

### Spatial heterogeneity in stress magnitude, not stress anisotropy, is the mechanical cause of cell elimination

**Fig 6:**
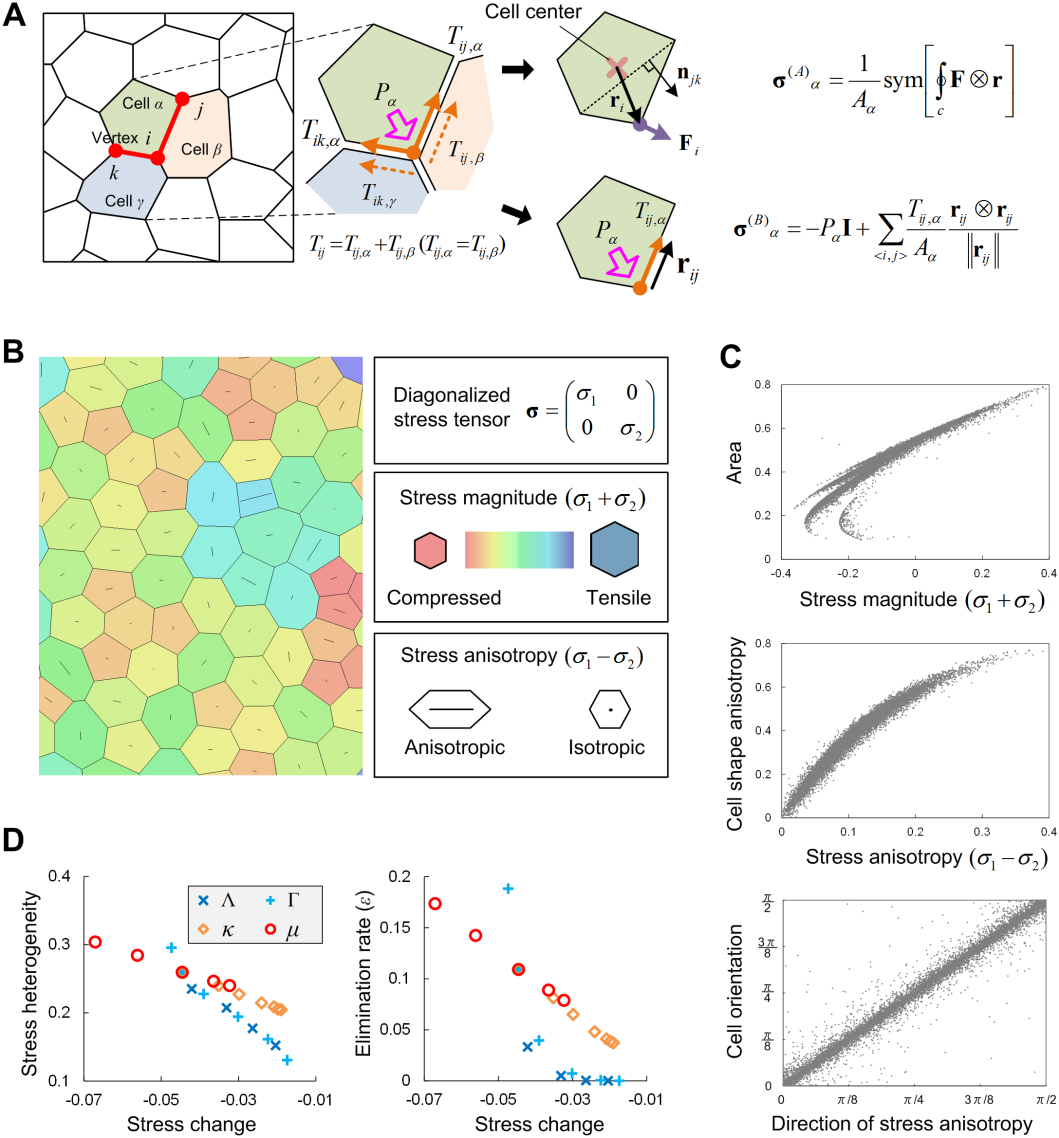
Spatial heterogeneity in stress magnitude as the mechanical cause of cell elimination. (A) The stress within a tissue was evaluated by two types of stress tensors that are discrete versions of Cauchy’s stress and defined by using the forces acting on the vertices that compose each polygonal cell. The forces acting on vertex *i* of cell *α* are composed of the pressure inside the cell, *P*_*α*_, and tension at the two edges linked to the vertex, *T*_*ij*,*α*_, *T*_*ik*,*α*_. The edge tension involving cell *α* was assumed to be half of the tension acting on the focal edge, *T*_*ij*_, and the remaining half was allotted to the other cell *β* that shares the edge, i.e., *T*_*ij*_ = *T*_*ij*,*α*_+*T*_*ij*,*β*_ = 2*T*_*ij*,*α*_. The stress tensor ***σ***^(*A*)^_*α*_ given by Eq (25) was calculated using the force vector **F**_*i*_, positional vector **r**_*i*_ from cell center, and normal vector **n**_*jk*_ (right upper). The stress tensor ***σ***^(*B*)^_*α*_ given by Eq (27) was calculated using the pressure *P*_*α*_, identity matrix **I**, tension *T*_*ij*,*α*_, and positional vector **r**_*ij*_ from focal vertex *i* to the adjacent one *j* (right lower; also see the Models section). (B) Stress magnitude, stress anisotropy and its orientation. The calculated tensors were characterized by the two scalars, stress magnitude *σ*_1_+*σ*_2_ and stress anisotropy *σ*_1_-*σ*_2_, where *σ*_1_ and *σ*_2_ (*σ*_1_>*σ*_2_) are the principal stresses. The left panel shows an example of stress distribution. The color indicates stress magnitude: blue for tensile states and red for compressed states. The line inside each cell indicates the degree of stress anisotropy and its orientation. Longer lines show more anisotropic states. The orientation is the direction of the maximum principal stress. (C) Correlations between the stress state and cellular geometry. Relationship between stress magnitude and cell area (top). Relationship between stress anisotropy and cell shape anisotropy (middle). Relationship between directions of stress anisotropy and cell shape anisotropy (bottom). Each graph is composed of 10,000 data points calculated from cells randomly selected at a specific time in one simulation run. Parameter values: the reference set, Λ = 0.14, Γ = 0.04, *θ*_T1_ = 0.1, *κ* = 0 and *μ* = 3.47×10^−3^. Correlation coefficient: *ρ*≈0.94 (top), *ρ*≈0.97 (middle), *ρ*≈0.99 (bottom). (D) Good correlation between the mean change in local stress magnitude and stress heterogeneity over the tissue (left) or the elimination rate (right). All simulations were performed using a pure population. Symbols: blue crosses (Λ), cyan pluses (Γ), orange diamonds (*κ*), and red circles (*μ*).

Since stress in a tissue drives its deformation, we next evaluated the stress state acting on each cell in a growing tissue. In this study, tissue is modeled not as a continuum but as a multicellular assembly, and thus Cauchy’s stress acting on each cell was evaluated by its microscopic and discrete representation. Among different representations hitherto proposed [44,45], we here adopted the two types of stress tensors used in recent papers on stress distribution in developing tissues [46,47] (see ***σ***^(*A*)^ and ***σ***^(*B*)^ in Fig 6A, and the Models section for details). The calculated tensors were characterized by the two scalars, stress magnitude *σ*_1_+*σ*_2_ and stress anisotropy *σ*_1_-*σ*_2_, where *σ*_1_ and *σ*_2_ (*σ*_1_>*σ*_2_) are the principal stresses (Fig 6B). Positive (or negative) values of *σ*_*i*_ represent a tensile (or compressive) stress. Correlation analysis with cell geometry showed that the stress magnitude and stress anisotropy are strongly correlated (>0.9) with cell size and cell shape anisotropy, respectively (Fig 6C, top and middle). This holds for cases with different mechanical/growth parameters and for either definition choice for stress tensor (Eqs (25) and (27) in the Models section). With regard to stress anisotropy, its direction (defined as the direction of the maximum principal stress) is perfectly consistent with the direction of cell shape anisotropy (Fig 6C, bottom). In this manner, in a pure population, cell geometry perfectly reflects the stress state acting on it. As seen later, when growth/mechanical properties vary through mechanical feedback or when a tissue is composed of cells with different properties, the correlation between cell geometry and stress state decreases somewhat (around 0.7).

We showed in the previous section that the cell elimination rate is determined by the variance in cell size or cell density. Thus, we can conclude that the spatial heterogeneity of stress magnitude, not that of stress anisotropy, is the main mechanical cause of cell elimination. To avoid misunderstandings, we emphasize that the heterogeneity described here is not driven by the difference in mechanical/growth properties between cells. Rather, in all simulations in Fig 6, all cells had the same parameter values (i.e., a pure population). The stress heterogeneity observed here is intrinsic to growing tissues.

To clarify the relationship between the heterogeneity of stress magnitude and tissue growth by cell division, we examined the change in stress states experienced by cells surrounding each dividing cell under different mechanical/growth rules. In all cases, the stress magnitude always decreased (became more compressed) on average. As expected, the average local stress change,

$$\text{E}[{\left(\sigma_{1}+\sigma_{2}\right)}_{After\;division}-{\left(\sigma_{1}+\sigma_{2}\right)}_{Before\;division}],$$ (4)

correlates well with the spatial heterogeneity in stress magnitude, and consequently with the cell elimination rate (Fig 6D). We also examined the change in local stress through the cell elimination event. In contrast to the case of cell division, cell elimination caused a release in stress on average. This suggests that MCE can be a mediator for homogenizing the tissue stress state (i.e., stress homeostasis) as well as actomyosin activity. This result is consistent with observations from previous experimental studies in which a potential role for cell elimination in the maintenance of homeostasis in epithelial tissues was shown [4,13]. Similar to the case of cell division, cell elimination only affected stress magnitude, not stress anisotropy.

**Fig 7:**
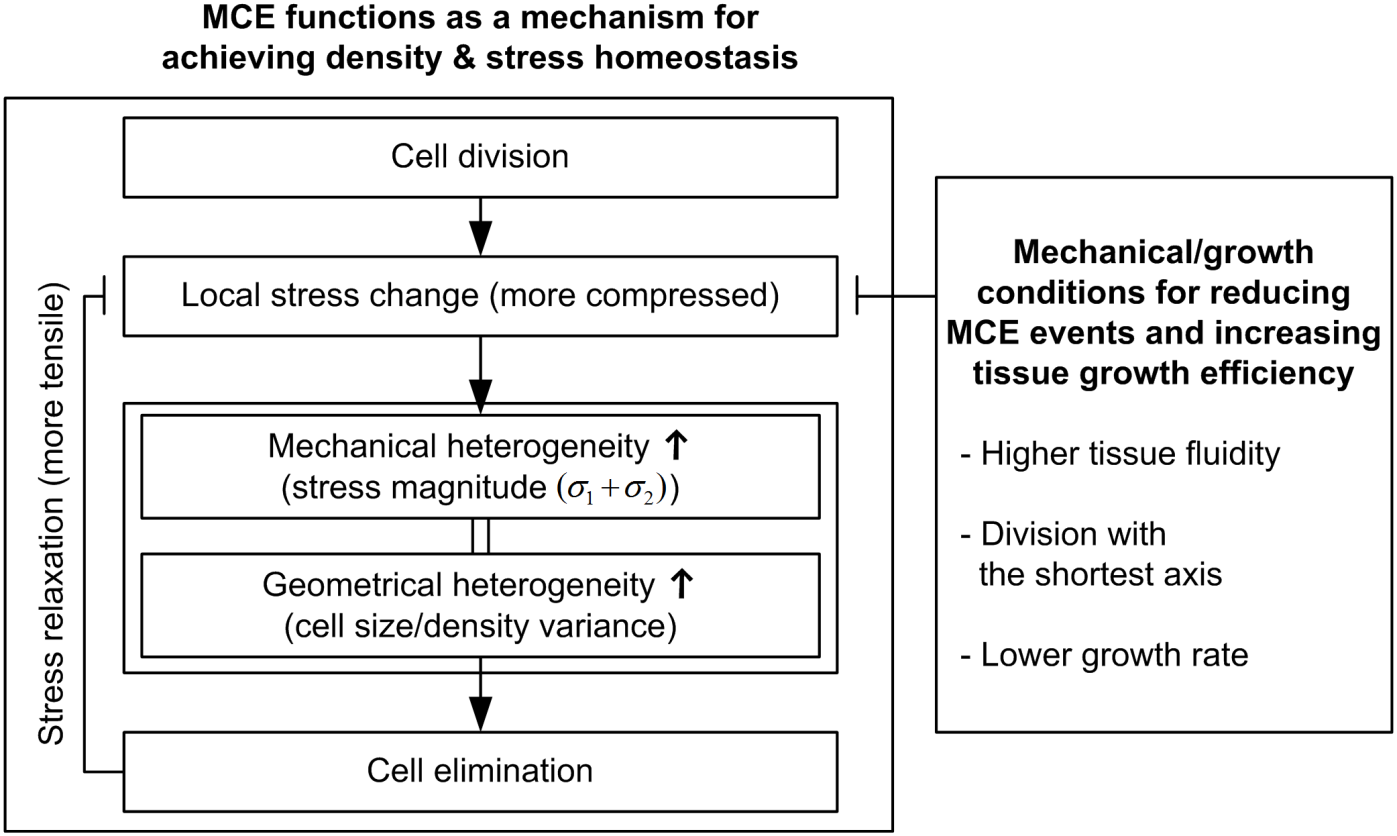
Summary of the results obtained with a pure population in the 2nd-4th subsections. During tissue growth, cell divisions induce surrounding tissue compression, and this results in an increase in the spatial heterogeneity of stress magnitude and cell size. As the cell size variance increases, more cells are eliminated from the tissue. In turn, cell elimination releases the compression due to cell division. From the viewpoint of energy efficiency, this process should be reduced and more newly-born cells should contribute to tissue growth; this can be achieved by higher tissue fluidity, division along the shortest axis, and lower growth rate.

Fig 7 shows a summary of the results obtained with a pure population in the above (2nd-4th) subsections. When tissue grows, cell division induces surrounding tissue compression, which increases the spatial heterogeneity in stress magnitude and cell size/density. The heterogeneity of cell size/density (or stress magnitude) is the common geometrical (or mechanical) trigger of MCE. Once cell elimination occurs, the compression due to cell division is released and the variance in local cell density also decreases. Thus, MCE functions as a mechanism for achieving density and stress homeostasis. On the other hand, from the perspective of energetic efficiency, reducing MCE events and increasing the contribution of newly-born cells to tissue growth can achieve target size with less energy resources, which can be achieved by higher tissue fluidity, division along the shortest axis, and lower growth rate.

### Possible mechanical feedback mechanisms that could improve tissue growth efficiency and homeostasis

In the above sections, to clarify the effects of cellular mechanical/growth parameters on the elimination rate or the loss of energy in the fitness function (see the first subsection), we assumed a pure population in which all cells had the same values for mechanical/growth parameters. However, in actuality, the values of these parameters can change among cells even if all of them have an identical genetic background. For example, cells can change their physical properties and/or growth rate through feedback depending on the stresses acting on them or mechanical environment. In addition, due to various noise sources such as intrinsic fluctuations in gene expression levels and extrinsic environmental noise [48,49], the parameter values can show a distribution among the cells. Here we examine the possibility of improving tissue growth efficiency (or tissue fitness *ϕ*_*Tissue*_) and homeostasis through mechanical feedback; in the next section we will examine the results of competition in a population where cells with different mechanical parameters are mixed.

**Fig 8:**
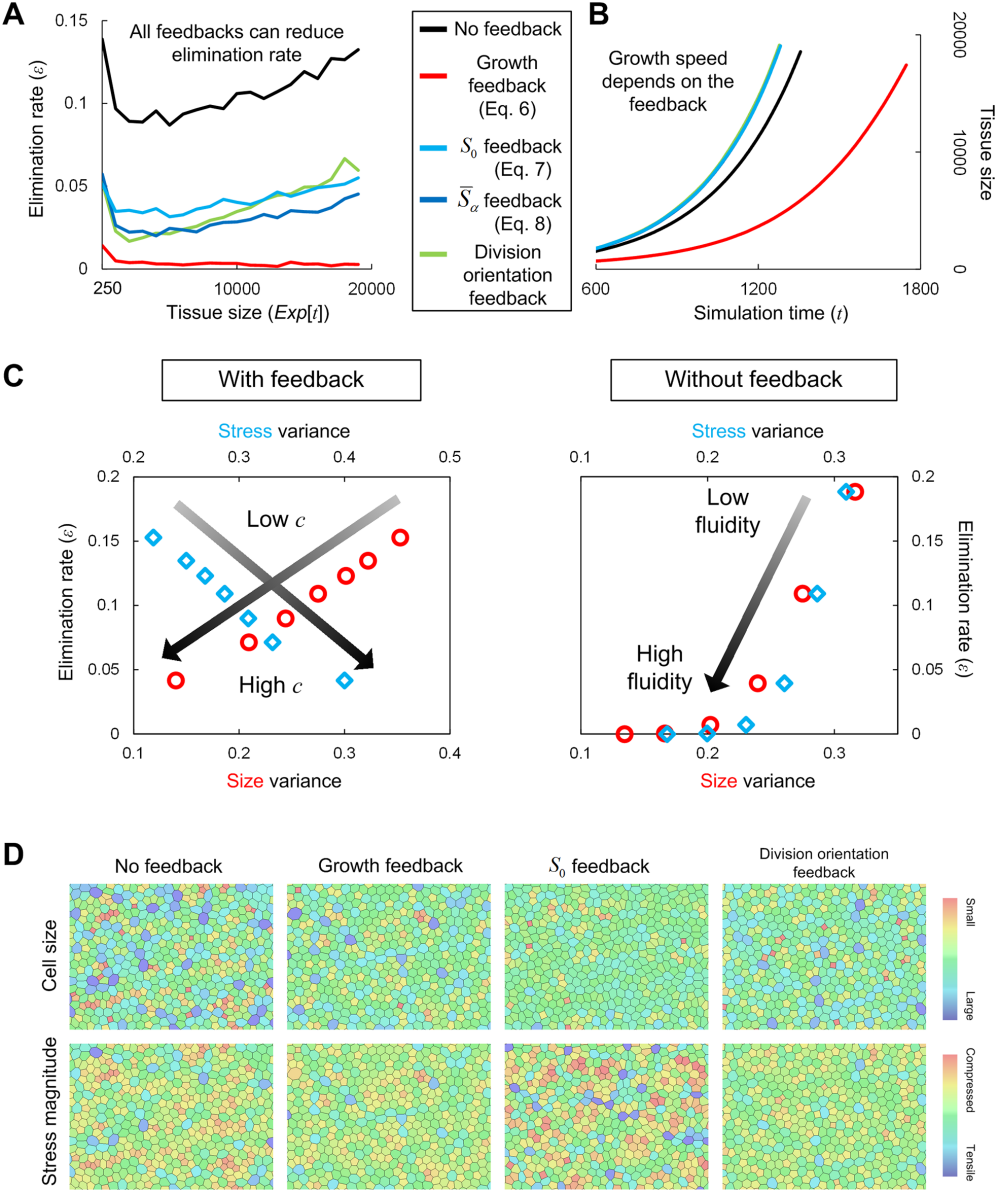
Possible mechanical feedback mechanisms that could improve tissue growth efficiency and homeostasis. (A) Time evolution of the elimination rate *ε* with and without mechanical feedback mechanisms. All feedback mechanisms outlined in the text significantly reduced the elimination rate *ε*. (B) Time evolution of tissue size with and without mechanical feedback mechanisms. Although the density-dependent growth regulation (red line) leads to a drastic decrease in the elimination rate, it takes much more time to attain a certain tissue size (e.g. 20,000 cells) compared to cases without feedback (black line), meaning that it was not necessarily efficient in regard to developmental speed. In contrast, the other feedback mechanisms, including feedback regulation of tissue fluidity given by Eqs (6) and (7) (cyan and blue lines), and that of cell division orientation (green line), could improve tissue growth efficiency by reducing the elimination rate and by maintaining a normal growth speed. (C) (Left) Incompatibility between the reductions in cell size variance and stress magnitude under a feedback model of tissue fluidity (Γ) given by Eq (6). When the feedback strength *c* is positive and larger, the cell size/density variance and elimination rate decreased but the variance in stress magnitude increased (see also (D) for an example of the simulation results). In contrast, when *c* is negative and smaller (i.e., |*c*| is larger), the opposite is true; although the cell size/density variance and elimination rate increased, the stress state was homogenized. (Right) For comparison, the relationship between the elimination rate and variance in cell size or stress magnitude is shown in the cases without feedback. The variances and elimination rate are lower for higher tissue fluidity, and vice versa. Graph symbols: red circles indicate the variance in cell size; blue diamonds indicate the variance in stress magnitude. Parameter values: (left) *c* ranges from -0.004 to 0.004. (right) Γ ranges from 0.02 to 0.05. (D) Examples of the spatial distribution of cell size (upper) and stress magnitude (lower) in the presence and absence of mechanical feedback; from the left: without feedback, with density-dependent growth regulation, feedback to tissue fluidity, and feedback to cell division orientation. All feedbacks could reduce the variance in cell size and density, but in the case of the feedback to tissue fluidity, there was incompatibility between the reductions in variances of cell size and stress magnitude. For the other two feedback mechanisms, the both were compatible.

Density- or stress-dependent growth regulation is a type of feedback that has been discussed extensively [29–31], although the molecular mechanism of mechano-sensing is not entirely clear. As a plausible example, we first examine how this feedback would affect *ϕ*_*Tissue*_. In particular, we modeled the clock of the cell cycle *τ*_*α*_ as a function of stress magnitude *S* = *σ*_1_+*σ*_2_:

$$\frac{d}{dt}\tau_{\alpha }(t)=\{\begin{array}{cc} 0 & \left(S<\bar{S}\right)\\ \text{const.} & \text{(}S\geq \bar{S}\text{)} \end{array},$$ (5)

where $\bar{S}$ is the mean stress magnitude before tissue growth starts. As mentioned before, when the clock becomes larger than the threshold *T*, cell division occurs. This stress-dependent growth regulation led to a large decrease in the elimination rate (Fig 8A) by preventing spatial heterogeneity in cell density, clearly demonstrating that this type of feedback can promote both tissue growth efficiency and homeostasis. However, it was not necessarily efficient in the sense of developmental speed, as it took much more time to reach a certain tissue size compared to cases without feedback (Fig 8B).

Developmental speed is an evolutionarily significant trait as well as growth efficiency and homeostasis. Our results in the previous sections suggest another possible means by which mechanical feedback could improve tissue growth efficiency and homeostasis: it could decrease the elimination rate while maintaining the growth speed. Here, we assume that cells can sense their own stress state or that of adjacent cells through their cytoskeletons or filopodia [50–52] and that, depending on the state, they can change local tissue fluidity by appropriately regulating their contractility and/or edge tension (i.e., Γ and Λ in the vertex dynamics model) through a change in intracellular localization of actin and adhesive molecules. As shown above, the elimination rate perfectly correlates with the variance in cell size or that of stress magnitude, and thus the proposed feedback mechanisms must be designed to decrease either of them. Specifically, we considered the following feedback systems;

$$\frac{d}{dt}\chi_{\alpha }(t)=c(S_{\alpha }-S_{0})-d(\chi_{\alpha }(t)-\chi^{0})$$ (6)

or

$$\frac{d}{dt}\chi_{\alpha }(t)=c(S_{\alpha }-{\bar{S}}_{\alpha })-d(\chi_{\alpha }(t)-\chi^{0}),$$ (7)

where *χ*_*α*_(*t*) is the parameter for apical contractility or cell-cell edge tension of cell *α* (i.e., *χ*_*α*_(*t*) = Γ_*α*_(*t*) or Λ_*α*_(*t*)), and *χ*^0^ (Γ^0^ or Λ^0^) is a cell-independent basal value. The magnitude of *c* indicates the feedback strength and *d* is the parameter determining the timescale of restoration to the basal value. When the value of *c* is positive, the feedback reduces cell size variance, whereas a negative value for *c* reduces the variance in stress magnitude. In the mechanism given by Eq (6), the apical contractility or cell-cell edge tension of a cell is regulated according to the difference of the stress magnitudes acting on the focal cell *α* from a reference value *S*_*α*_—*S*_0_. In contrast, if cells can sense the stress state of neighboring cells as well as their own state, it is possible there could be another feedback mechanism dependent on the difference from the average of adjacent cells $S_{\alpha }\;-\;{\bar{S}}_{\alpha }$, given by Eq (7).

The blue lines in Fig 8A and 8B show the results from growth simulations with either the feedback mechanism given by Eq (6) or (7). These mechanisms basically had the same effects on growth efficiency, i.e. elimination rate and growth speed. Importantly, we found that the reductions in cell size variance and stress variance were incompatible; positive and larger values of *c* decreased cell size variance (improved density homeostasis) but increased the stress variance (impaired the stress homeostasis), and vice versa (Fig 8C and 8D). As mentioned before, in the absence of feedback, the elimination rate is strongly correlated with the variance in cell size and stress magnitude. In the presence of the feedback, however, the elimination rate was negatively correlated with the variance in stress magnitude due to the incompatibility, although it is positively correlated with cell size variance (Fig 8C). In regard to the growth speed, the feedback with different strengths *c* returned almost the same value (Fig 8B). Consequently, to improve growth efficiency and density homeostasis by reducing the elimination rate and by keeping the growth speed normal, it is desirable that the parameter *c* in Eqs (6) and (7) has a positive value with which the feedback mechanism can reduce cell size variance. In contrast, the simulation results also shows that feedback with a negative value of *c* can achieve stress homogenization by actively inducing cell elimination.

Lastly, we considered the feedback to cell division orientation. As shown in Fig 3A, cell division along the shortest axis of a cell significantly suppressed the elimination rate. Since stress anisotropy perfectly correlates with cell shape anisotropy (Fig 6C), by reflecting the stress anisotropy to spatial arrangement of centrosomes that determine the division axis, tissue growth efficiency and density homeostasis could be improved. Importantly, through this feedback, the reductions in cell size variance and stress variance are compatible. This mechanism looks relatively simple, but it is an excellent way to improve tissue growth efficiency and maintain geometrical and mechanical homogeneity in a growing tissue. In the context of plant development, Alim et al. discussed the relationship between stress variability and division orientation [46].

### Cell competition through MCE can improve tissue growth efficiency and homeostasis through the selection of mechanical cell traits

**Fig 9:**
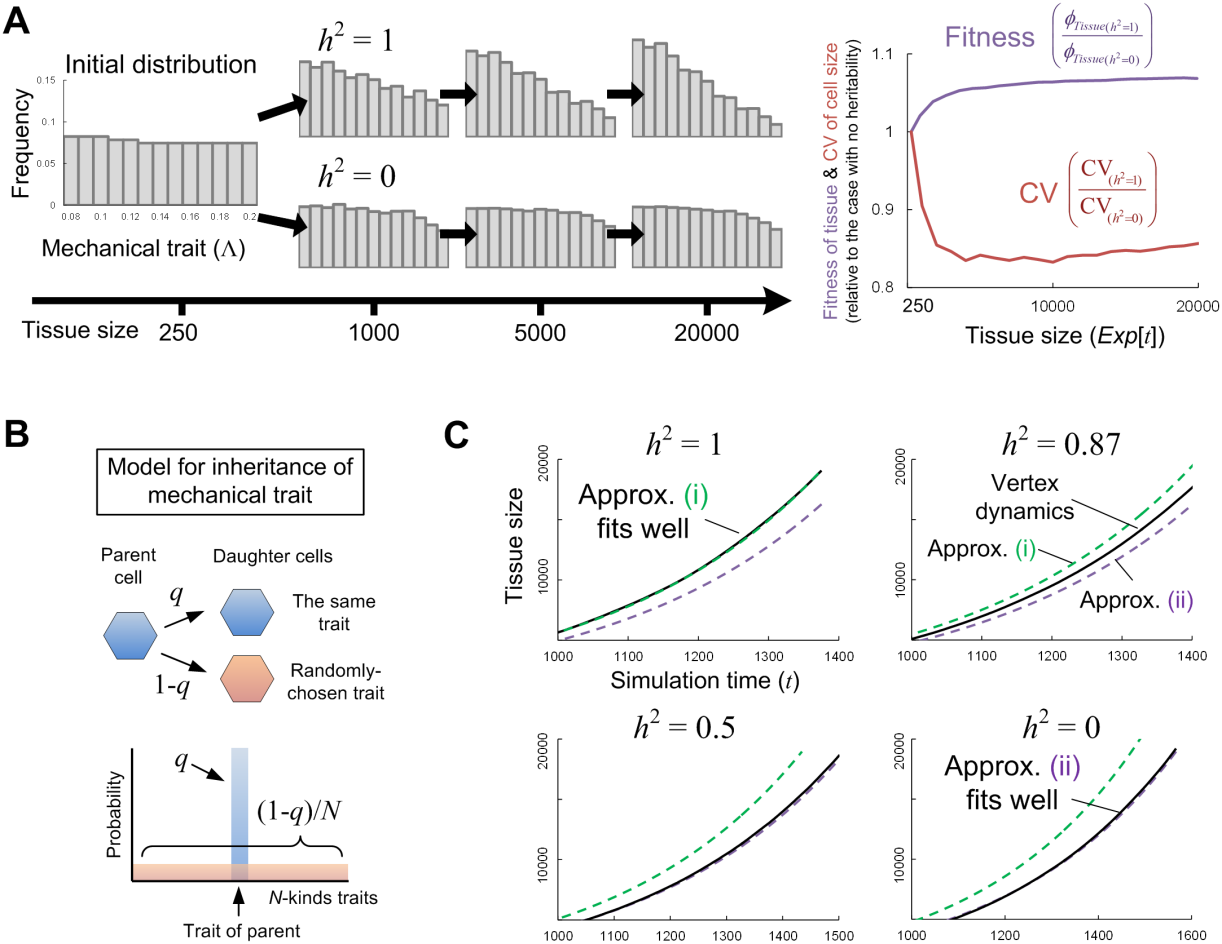
Improvement in tissue growth efficiency and homeostasis over time with cell competition through MCE in a mixed population. (A) A simulation result for the time evolution of frequency distribution for a mechanical cell trait. In the case of perfect inheritance of the trait (i.e., *h*^2^ = 1), the frequency distribution drastically changes (left). At the same time, tissue-level fitness and density homeostasis are improved through tissue growth (right). In other words, intra-tissue evolution can occur. In contrast, in the case of no inheritance (i.e., *h*^2^ = 0), the frequency distribution of a cellular trait, the tissue fitness, and density homeostasis never changes. (B) Schematic diagrams of a model for inheritance of a mechanical trait. The trait of a daughter cell is inherited from its parent (blue) with probability *q* and is uniformly and randomly chosen from all the possibilities (red; among a discrete value of *N*-kinds) with probability 1-*q* (thus, *q* = 1 for perfect inheritance and *q* = 0 for no inheritance). (C) Growth curves for different values of heritability. The black solid lines are the curves obtained by simulations of vertex dynamics model. The green dashed lines show the growth curves obtained by the approximation (i), which is effective in cases with much higher heritability where each cell population of each trait can be regarded to grow independently (see Eq (9)). The purple dashed lines show the growth curves by the mean field approach (approximation (ii)), which is effective in a case with lower heritability (see Eqs (8)–(13)).

Even in genetically identical cell populations, phenotypic variations in cellular traits (mechanical/growth properties) are inevitable. Here, we examined the results of mixing cells with different traits. For example, as an extreme situation let us consider two cases where (i) daughter cells perfectly inherit parental traits and (ii) traits of daughter cells are randomly distributed. In terms of evolutionary theory, the former is a case in which heritability *h*^2^ = 1, and the latter *h*^2^ = 0. Fig 9A shows the temporal evolution of frequency distribution ***f***(*t*) = (*f*_1_(*t*), …, *f*_*N*_(*t*)) of cells with different values of a mechanical trait, Λ or Γ, where all cells have the same cell cycle time. In the case of *h*^2^ = 1, the distribution drastically changed with tissue growth and, as a result, the fitness increases at the tissue level (see Eq (2) for its definition; right panel of Fig 9A), clearly demonstrating that cell competition through MCE can improve tissue growth efficiency through the selection of mechanical cell traits. At the same time, cell size and stress were homogenized during growth (the right panel of Fig 9A). In contrast, when a trait is not inherited from a parent (i.e., *h*^2^ = 0), the frequency distribution of the cellular trait and tissue fitness never change, even though cells with traits giving higher cellular fitness survive.

How do the trait distribution ***f***(*t*) and the tissue-level fitness *ϕ*_*Tissue*_ change when tissue size becomes much larger, e.g. more than 10^6^ cells (fully-developed size), or when the heritability *h*^2^ takes a value other than 1 and 0? Since directly simulating the vertex dynamics model requires an immense amount of computation time to generate such a huge number of cells, we sought a way to calculate the approximate time evolution of the competition process. To do so, we first modeled the process of inheriting the mechanical traits from the parental cell. Let us suppose that the focal trait has a discrete value of *N*-kinds and that the trait of the daughter cells is inherited from its parent with probability *q* and is uniformly and randomly chosen from all the possibilities with probability 1-*q* (Fig 9B). Then, the heritability *h*^2^ monotonously changes with the value of *q* (where *q* = 1 indicates perfect inheritance and *q* = 0 no inheritance). The black lines in Fig 9C show examples of tissue growth curves for different values of *h*^2^.

The time evolution of the number of cells with each trait, *x*_*i*_(*t*) (*i* = 1, …, *N*) is given by the following ordinary differential equation:

$$\frac{dx(t)}{dt}=Q_{q}\Phi_{cell}(t)x(t),$$ (8)

where ***x***(*t*) = (*x*_1_(*t*), …, *x*_*N*_(*t*)). *Q*_*q*_ is the matrix relevant to the heritability whose diagonal and off-diagonal components are *q*+(1-*q*)/*N* and (1-*q*)/*N*, respectively. Φ_*Cell*_(*t*) is the matrix whose *i*-th diagonal component is the cellular fitness of the *i*-th trait *ϕ*_*Cell*,*i*_(*t*), and all of the off-diagonal components are zero.

**Fig 10:**
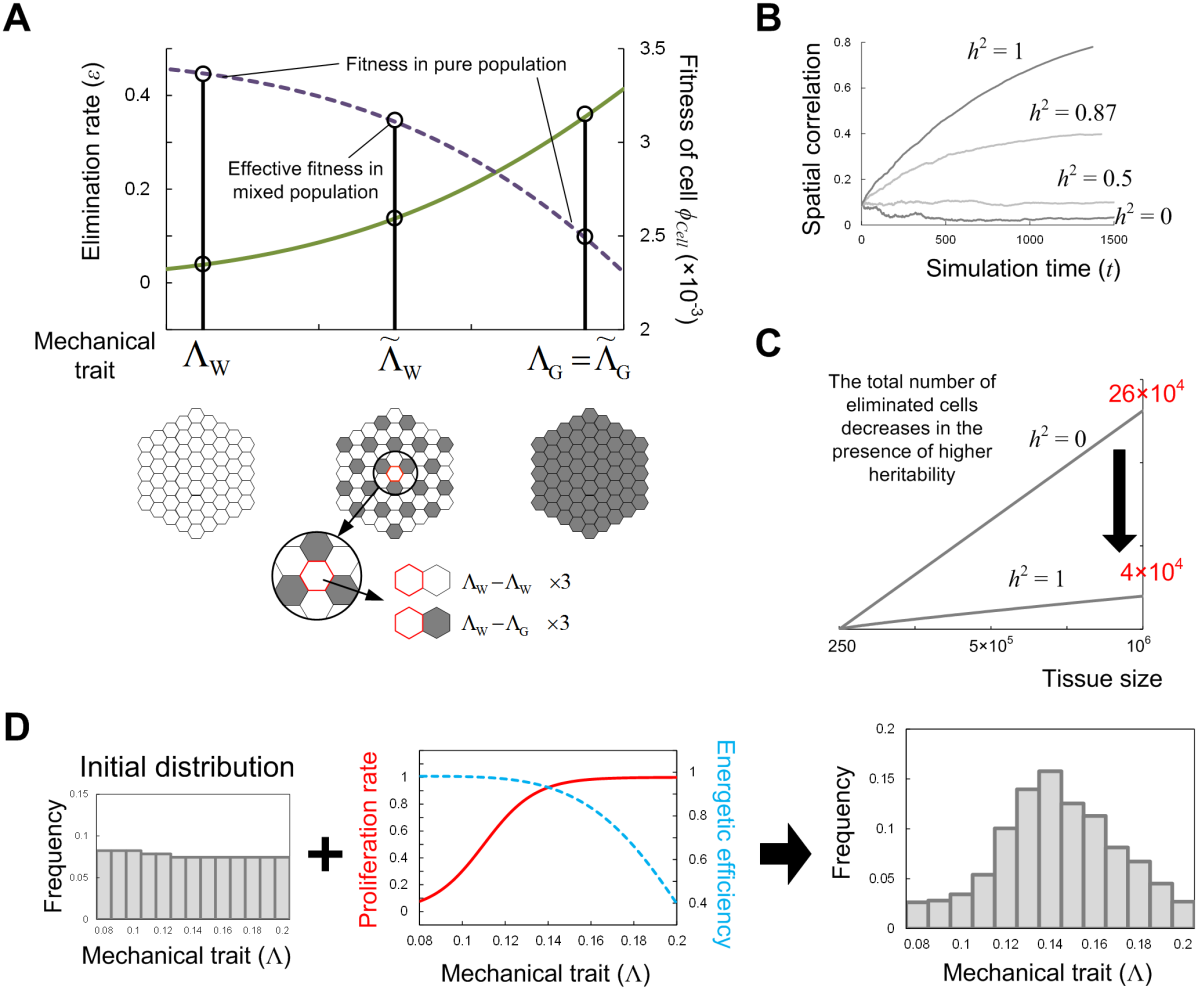
Approximating the dynamics of trait distribution. (A) Schematic diagrams of the calculation for effective fitness. In a pure population, each cell has an elimination rate (solid green line) and cellular fitness (dashed purple line) determined according to its own mechanical trait, e.g., the coefficient of line tension (Λ_W_ and Λ_G_, W and G indicate the white cells and gray cells, respectively). In contrast, in a mixed population, the elimination rate of each cell depends on the mechanical properties of adjacent cells. Suppose that each cell makes contacts with cells of the same or different kind with equal probability. Then, when the effective coefficient of line tension of an edge is modeled by max(Λ_W_, Λ_G_) (see text) and the inequality Λ_W_ < Λ_G_ is assumed, the effective value is ${\tilde{\Lambda }}_{W}=(3\Lambda_{W}+3\Lambda_{G})/6$ for the white cells and ${\tilde{\Lambda }}_{G}=\Lambda_{G}$ for the gray cells. Using these effective trait values, the fitness of each cell can be calculated as $\phi_{Cell}({\tilde{\Lambda }}_{W})$ and $\phi_{Cell}({\tilde{\Lambda }}_{G})$, where *ϕ*_*Cell*_ is the function of cellular fitness obtained in Fig 3A. (B) Time evolution of the spatial correlation of mechanical cell traits. We used Moran’s *I* for the index of spatial correlation. For higher heritability, the correlation was higher, meaning that cells with the same trait (i.e., descendant cells) tended to easily form clusters, for which the approximation (i) works well. In contrast, for lower heritability, the correlation was lower, and cells with different traits would be well mixed, for which approximation (ii) works well. (C) Time evolution of the total number of eliminated cells estimated by the proposed approximation method in a case with much larger tissue size. When the number of cells within a tissue reaches 10^6^, the total number of eliminated cells through tissue growth was 4×10^4^ in the case of *h*^2^ = 1 (obtained by approximation (i)), while 26×10^4^ for *h*^2^ = 0 (obtained by approximation (ii)). This demonstrates that cell competition through MCE, especially in earlier phases of development, is able to considerably reduce energy loss and improve tissue growth efficiency in the presence of high heritability. (D) Frequency distribution of a mechanical trait could evolve so that it has an intermediate peak value when tissue fluidity affects both cell elimination and proliferation rates (right). (Middle) Specifically, the cell proliferation rate was given as a monotonously increasing function of Λ (solid red line; 1/(1+*Exp*[-10Λ])). The blue dashed line shows the energy efficiency (1-*m*/*μ*), and *h*^2^ = 0.87 was adopted. In actuality, mechanical cell traits are expected to be homogenized through tissue growth under different tradeoffs.

In contrast to the pure population case examined in the previous sections, the cellular fitness *ϕ*_*Cell*,*i*_ is generally not constant but changes with tissue growth as well as tissue-level fitness *ϕ*_*Tissue*_. This is because the elimination rate of each cell depends on mechanical properties of adjacent cells. For example, when the coefficient of line tension Λ on a cell edge is regulated by the amount of homophilic adhesive molecules and its value is different between two contacting cells, cell *α* and cell *β*, the effective value $\tilde{\Lambda }$ of the parameter on the edge is regarded as max(Λ_*α*_, Λ_*β*_) or simply approximated by the average (Λ_*α*_ + Λ_*β*_)/2 (Fig 10A; also see the Models section). This makes the elimination rate different from cases with a pure population.

Exceptionally, when the heritability is sufficiently large, cells with the same trait form a cluster, and thus *ϕ*_*Cell*,*i*_ can be approximated as a constant value calculated in the pure population case (see Fig 9C for cases where *h*^2^ = 1, and Fig 10B for the spatial correlation of the cell trait). In this case, Eq (8) becomes a linear differential equation and thus can be solved analytically (approximation (i)):

$$x(t)=Exp[Q_{q}\Phi_{cell}t]x(0).$$ (9)

Using the frequency distribution,

$$f(t)=x(t)/\sum_{i}x_{i}(t),$$ (10)

the time evolution of tissue fitness can be calculated from Eq (2). Fig 9C shows that this approximation (i) works well.

In contrast, when the heritability is not high, it is necessary to adjust mechanical parameters (and thus the cellular fitness value). For example, when Λ is adjusted by the max-function max(Λ_*α*_, Λ_*β*_), the mean of its effective value for cells with the *i*-th trait is given as follows using the mean field approach,

$${\tilde{\Lambda }}_{i}(t)=\Lambda_{i}\sum_{k=1}^{i}f_{k}(t)+\sum_{k=i+1}^{N}\Lambda_{k}f_{k}(t),$$ (11)

where we assume Λ_*i*_ < Λ_*j*_ for *i* < *j* without loss of generality. Then, *ϕ*_*Cell*,*i*_(*t*) can be estimated by using the function for the elimination rate *ε*(Λ) obtained in the second subsection (Figs 3A and 10A):

$$\phi_{Cell,i}(t)=\text{log}\left(2-\varepsilon ({\tilde{\Lambda }}_{i}(t))\right)/T.$$ (12)

Finally, by numerically solving a set of equations, Eqs (8) and (10)–(12), the time evolution of ***x***(*t*), ***f***(*t*), ${\tilde{\Lambda }}_{i}(t)$ and *ϕ*_*Cell*,*i*_(*t*) was obtained (approximation (ii)). Fig 9C shows that this solution is in good agreement with the results of a full simulation with the vertex dynamics model. As a special case, when the heritability is sufficiently small, *h*^2^ ≈ 0, since the trait distribution is almost uniform. Thus, *f*_*i*_(*t*) ≃ 1/*N*, Eq (10) can be approximated as follows:

$${\tilde{\Lambda }}_{i}(t)≅\frac{1}{N}(i\Lambda_{i}+\sum_{k=i+1}^{N}\Lambda_{k})=\text{const.}$$ (13)

In this case, the cellular fitness *ϕ*_*Cell*,*i*_ becomes constant (see Eq (12)) and thus, as in the other extreme case where *h*^2^ ≈ 1, Eq (8) becomes a linear equation and can be solved analytically. Again, Fig 9C shows the validity of this approximation.

Now, by using the above approximation approach, we can predict the time evolution of the trait distribution ***f***(*t*) and the tissue fitness *ϕ*_*Tissue*_ when the tissue size is very large. For example, when the number of cells within a tissue reaches 10^6^, the total number of eliminated cells through tissue growth was 4×10^4^ in the case where *h*^2^ = 1 and 26×10^4^ for *h*^2^ = 0 (Fig 10C). In this manner, cell competition through MCE can considerably reduce the loss of energy and improve tissue growth efficiency when heritability is high. Although there are few studies of multicellular organisms and the extent to which a daughter cell epigenetically inherits parental trait, such epigenetic inheritance is observed in the persistence phenomenon in bacteria [53]. Thus, a non-negligible degree of heritability is expected in the developmental processes of multicellular organisms.

Above, we considered the situation in which the value of a trait, i.e. fluidity, is included in a certain finite range. In the absence of such a restriction, does the distribution of a trait continue to shift so as to increase tissue fluidity? And as a result, does the tissue fitness become higher? The answer is affirmative under the above settings of growth simulation, but in actuality, it is negative because of other tradeoffs not included in the simulation. For instance, as shown above, cells with smaller values of Λ have higher fitness. The smaller value of Λ means stronger adhesion between cells. Since cells with higher adhesion tend to have lower proliferation rates, in actuality, the value of Λ might affect not only cell mortality through elimination but also the cell proliferation rate [54]. To observe this, we performed tissue growth simulations where the cell proliferation rate was given as a monotonously increasing function of Λ. As shown in Fig 10D, the frequency distribution shifted so that it peaks at an intermediate value of Λ, reflecting the tradeoff between the contributions to tissue growth rate *μ*(*t*) and energy efficiency (1-*m*(*t*)/*μ*(*t*)). In this manner, even if phenotypic variability exists in the initial phase of development, mechanical cell traits can be homogenized (i.e., homogenization of phenotype) by cell competition through MCE during tissue growth.

## Discussion

### Study summary and possible roles for mechanical cell elimination (MCE)

In this study, we examined mechanical cell elimination, an intrinsic phenomenon in growing tissues, in a genetically-homogeneous tissue. First, we proposed novel quantitative measures to evaluate fitness in cell competition phenomena at the cellular and tissue levels. In a pure population where all cells have identical parameter values, cellular fitness was shown to be uniquely determined for each set of mechanical/growth parameters, and the analysis of simulation results demonstrated that the dependence could be summarized by how those parameters affect geometrical (specifically the variance in cell size) and mechanical (variance in stress magnitude) heterogeneity within tissues. This information has provided the basis for feedback regulation for improving tissue growth efficiency and homeostasis. Moreover, we showed that in the presence of phenotypic variation in mechanical cell properties, the distribution of a mechanical trait can shift with tissue growth depending on the heritability of a focal trait, resulting in the improvement of tissue growth efficiency and homeostasis. In addition, from a more mathematical perspective, we developed a theoretical model to approximate the time evolution of trait distribution and fitness. This model enabled us to predict the outcomes when tissue size was much larger without performing direct simulations of the cell-based model that would require an immense amount of computation time.

Our results clearly demonstrate the potential roles for MCE in tissue growth efficiency and homeostasis. One role is to promote geometrical and mechanical homogeneity of tissues by removing the cells in which stress and strain are concentrated due to cell division (Fig 7). This role as a homogenizer itself has also been discussed in previous experimental studies [4,13,15]. We quantitatively confirmed this homogenization process (Fig 5D), and also showed that the role as a homogenizer can be improved with appropriate regulation of mechanical/growth parameters depending on cellular stress state. Running the simulations in the presence of phenotypic variation, we showed that MCE could improve tissue growth efficiency by selecting cells with higher fitness, which is another potential role for MCE. This is important in an energetic sense. The energy required for tissue growth is proportional to the number of cells that are produced. Thus, improving tissue growth efficiency through competition during an earlier phase of development (when tissue size is still small) would provide a great advantage as the tissue increased in size. Along with the improvement of tissue growth efficiency, in the presence of phenotypic variation, MCE also homogenized or autonomously tuned cellular phenotype (mechanical parameters), as well as homogenization of cell density and stress state, through tissue growth. According to studies of the vertex dynamics model [31,43,55], there is a limited range of mechanical parameters that can reproduce some of the laws observed in experiments such as Lewis’s law (the relationship between cell size and the number of angles in a polygon representing a cell) and responses to mechanical stimuli [35,56,57]. Our result shows a possible mechanism by which cells could tune the values of their parameter. That is, MCE in growing tissues could select cells with desirable parameter values.

MCE in a mixed cell population with phenotypic variation can be regarded as a specific case of cell competition between populations of cells with different genetic backgrounds. Although specific signaling pathways that induce apoptosis and phagocytosis have been examined [7,8,37], the shrinkage of less fit cells through mechanical cell-cell interaction can be one of the first cues that induce those events, and the elimination of the cell would be supported by additional feedback regulation. As stated in the introduction, reports on MCE under genetically-heterogeneous conditions [10,15,21] support this possibility. If such a mechanical view is valid, as shown in Figs 9 and 10, the rate of elimination or cellular fitness would be determined by the difference in mechanical/growth parameters between cell populations and the frequency distribution of cells with different mechanical traits. This also suggests the possibility of promoting the elimination of a cell population (abnormal cells) and preventing tumor growth by appropriately regulating mechanical cellular properties through mechanical feedback. These will be important and interesting topics for future work.

### Potential for experimental verification

The parameter dependence of the MCE rate shown in Fig 3A would be verifiable with current experimental settings. For example, suppressing cell-cell adhesion and promoting the rate of cell division would be experimentally feasible by manipulating related molecules. Under such conditions, an increase in cell size variance and elimination rate would be expected. In addition, based on our simulation results, the spatial heterogeneity of stress magnitude (defined as the trace of the stress tensor) would also be expected to rise. Applying the methods of force inference from cellular geometrical information (i.e., vertex network) [47,58,59], the tension on a cell-cell edge and relative value of the intracellular pressure could be calculated, with which it would be possible to estimate the spatial heterogeneity in stress magnitude. According to the results shown in Fig 4A, when the cell size variance is somewhat large (CV>0.2 for our simulation settings), the CV for cell size and the elimination rate are expected to have an almost linear relationship with changes in the different parameters, although the lower limit value for the linearity (i.e., CV = 0.2) may depend on the value of *θ*_T2_. In this regard, however, it should be noted that we examined the responses (cell elimination rate) for situations where each parameter value was changed independently (Figs 3 and 4), although we do not know if this is experimentally possible or not. Furthermore, Fig 5B shows a high positive correlation between cell density and the CV for cell size, which could be verified in an artificially-densified cell culture system described in the introduction and/or in vivo systems such as *Drosophila* wing and notum development (by using the spatial difference in cell density).

As mentioned above, phenotypic variation between cells will naturally exist even in a genetically homogeneous cell population. As examined in the final subsection of the Results, in the presence of such variation, it should be possible to identify that cells with reduced expression levels of adhesive molecules and/or in which actomyosin activity is higher, are easier to eliminate, and that expression distribution would become more uniform. Again, these experiments would be possible in an artificially-densified cell culture system and/or in vivo systems such as *Drosophila* wing and notum development.

### MCE triggers

Although there are reports of MCE from both genetically-homogeneous tissues and genetically-heterogeneous tissues, there is little convincing evidence for any particular unambiguous cue that triggers elimination. In this study, we adopted cell area as a possible trigger for MCE based on the report by Marinari et al., which demonstrated in *Drosophila* notum development that cells with an area less than ~25% of their initial area were eliminated; to the best of our knowledge, this is the only report to quantitatively describe that trigger [4]. As shown in the 4th subsection of the Results, since cell size is highly correlated with the stress magnitude acting on a cell, our assumption on the criterion for MCE, i.e. the existence of a cell size threshold for MCE, also includes another potential criterion, the existence of a threshold for stress acting on a cell. Thus, our criteria for MCE include both geometrical and mechanical cell quantities, which we think are not so specific. On the other hand, Marinari et al. also reported a correlation between cell elimination and cell shape anisotropy during notum closure in *Drosophila* development, suggesting that a combination of multiple factors such as cell size and shape anisotropy (or the combination of stress magnitude and anisotropy), could be another candidate trigger for MCE.

### Factors other than growth efficiency that affect tissue-level fitness

In order to quantify the degree of competition, we adopted net growth speed or growth efficiency as a measure of tissue-level fitness. However, considering developmental processes, the spatial order between cells is also an important factor directly affecting the performance of pattern formation and tissue morphogenesis. For example, our results showed that the increase in tissue fluidity improves energy efficiency by suppressing cell elimination, but at the same time, spatial rearrangement between cells occurs frequently. This might make it difficult to realize robust tissue patterning. Thus, the frequency of cell rearrangement (quantified by the number of T1-processes) is a quantity that might be included in the fitness measure as well as tissue growth efficiency when considering patterning precision. Tamada et al. reported that in *Drosophila* axis elongation, the frequency of forming multicellular rosette structures is different in the presence or absence of the expression of Abl tyrosine kinase [41]. In addition, Levayer et al. showed that the efficiency of eliminating weaker cell populations is promoted by intermingling of cells with different genetic backgrounds [60]. These experimental studies suggest that the frequency of cell rearrangement or tissue fluidity would be controlled by cells. On the other hand, as mentioned before, the regulation of cell-cell adhesion is a possible mechanism to determine tissue fluidity. Adhesion and cell proliferation are related, and thus it would be generally impossible to control them independently. This means that growth speed and energy efficiency might be interdependent although we dealt with them as independent quantities in our model. In this manner, there are likely to be various tradeoffs in cellular/tissue behavior. In the future, by studying how cell competition contributes to cellular and tissue fitness in the presence of those tradeoffs, its biological significance will become clearer.

## Models

### Definition of tissue and cellular fitness in a mixed population

As given by Eq (1), in this study, we defined fitness at the tissue level, *ϕ*_*Tissue*_(*t*), by the time-derivative of the logarithm of the growth curve *g*(*t*) (*g*(*t*) is the total number of cells in a growing tissue at time *t*). As described in the first subsection of the Results, tissue fitness is generally a function of time, but in a pure population the growth curve can be well approximated by an exponential function with a constant exponent, *μ*-*m*, and thus tissue fitness is regarded as a constant, where *μ* is the tissue growth rate determined by the cell cycle time and *m* is the mortality determined by the rate of (mechanical) cell elimination. In contrast, in a mixed population, tissue fitness generally varies with time because the different cell types with different traits that comprise the entire tissue have different cellular fitness, and the net tissue growth rate depends on the frequency distribution of cellular traits at each time point. Denoting the number of cells with *i*-th trait at time *t* by *g*_*i*_ (*t*), the tissue size or the total number of cells comprising the tissue, *g*(*t*), is given by:

$$g(t)=\sum_{i=1}^{N}g_{i}(t).$$ (14)

Substituting this into Eq (1) which defines the tissue fitness, we obtain:

$$\phi_{Tissue}(t)=\frac{d}{dt}\text{log}g(t)=\frac{d}{dt}\text{log}\left(\sum_{i=1}^{N}g_{i}(t)\right)=\frac{1}{g(t)}\sum_{i=1}^{N}\frac{dg_{i}(t)}{dt}.$$ (15)

Using the frequency of cells with *i*-th trait, *f*_*i*_ *= g*_*i*_ (*t*)/*g*(*t*), this equation can be rewritten as:

$$\frac{1}{g(t)}\sum_{i=1}^{N}\frac{dg_{i}(t)}{dt}=\sum_{i=1}^{N}\frac{f_{i}(t)}{g_{i}(t)}\frac{dg_{i}(t)}{dt}=\sum_{i=1}^{N}f_{i}(t)\frac{d\text{log}g_{i}(t)}{dt}.$$ (16)

With the definition of cellular fitness with the *i*-th trait, *ϕ*_*Cell*,*i*_(*t*), given by Eq (3), the above equation is equivalent to Eq (2). Further, decomposing the cellular fitness at time *t* into the growth rate (*μ*_*i*_(*t*)) and mortality (m_*i*_(*t*)) of a cell population with *i*-th trait,

$$\frac{d\text{log}g_{i}(t)}{dt}=\mu_{i}(t)-m_{i}(t),$$ (17)

the tissue fitness can be written as:

$$\phi_{Tissue}(t)=\sum_{i=1}^{N}f_{i}(t)\mu_{i}(t)-\sum_{i=1}^{N}f_{i}(t)m_{i}(t).$$ (18)

Defining the tissue growth rate (*μ*(*t*)) and mortality (m(*t*)) by the first and second term on the right-hand side, respectively, the tissue fitness in a mixed population can be also represented as *μ*(*t*)- m(*t*).

### A relationship between mortality *m* and elimination rate *ε* in a pure population

As described in the first subsection of the Results, assuming that growth dynamics *g*(*t*) is modeled by *dg*(*t*)/*dt* = (*μ*(*t*)-*m*(*t*))*g*(*t*), the tissue fitness becomes *μ*(*t*)-*m*(*t*), where *μ*(*t*) is the tissue growth rate and *m*(*t*) is mortality. In a pure population, the fitness is roughly regarded as a constant and determined by the cellular processes, division and elimination. When all cells have the same value (on average) for cell cycle time *T* and cells are never eliminated (i.e. mortality *m* = 0), the number of cells rises 2^*N*^ -fold during the next *NT* hours (of course, the unit of time is arbitrary). Thus, fitting this cellular process to an exponential tissue growth curve, 2^*N*^ = exp(*μNT*) or *μ* = (log2)/*T* holds. Next, let us consider a case with cell elimination. Since the elimination rate is defined as *ε* = *N*_*eliminated*_/*N*_*produced*_, during *T* hours, the increment for the number of cells added by division (i.e. *N*_*produced*_) is one but a cell is eliminated at rate *ε*. As a result, the number of cells rises (2-*ε*)^*N*^ -fold over the next *NT* hours. Fitting these cellular processes to an exponential tissue growth curve, (2-*ε*)^*N*^ = exp((*μ*-*m*)*NT*) holds. Substituting *μ* = (log2)/*T* into this equation, we finally obtain the relationship *m* = -(log(1-*ε*/2))/*T*.

### A mechanical model of epithelial tissue growth

To simulate epithelial tissue growth, we adopted a vertex dynamics model (Fig 2), The vertex dynamics model has been used to study animal epithelial tissue growth, such as *Drosophila* wing disc, *Xenopus* tadpole fin, *Fundulus* epiboly [31,33–36] and plant tissue growth, such as shoot apical meristem and leaf development [61–63].

In the vertex dynamics model, each cell is represented as a polygon formed by linking several vertices, and the motion of each vertex is determined to decrease the energy function *U* of the system as follows:

$$\eta \frac{dr_{i}}{dt}=-\frac{\partial U}{\partial r_{i}},$$ (19)

$$U=\sum_{\alpha }^{}\frac{Κ}{2}{\left(A_{\alpha }-A_{0}\right)}^{2}+\sum_{<\alpha ,\beta >}^{}\Lambda_{\alpha \beta }l_{\alpha \beta }+\sum_{\alpha }^{}\frac{\Gamma_{\alpha }}{2}L_{\alpha }{}^{2},$$ (20)

$$\Lambda_{\alpha \beta }=\{\begin{array}{c} \text{ max}(\Lambda_{\alpha },\Lambda_{\beta })\\ \;(\Lambda_{\alpha }+\Lambda_{\beta })/2 \end{array},$$ (21)

where **r**_*i*_ is the positional vector of the *i*-th vertex and *η* is a coefficient of viscous resistance. The first term of *U* indicates an area constraint. If the polygonal area *A*_*α*_ of a cell with constant volume is changed, the cell height adjusts. Under such deformation, the elastic energy can be described by a coefficient Κ and a natural area *A*_0_. The second term represents the line tension and/or cell-cell adhesion on a cell edge *l*_*αβ*_ between cell *α* and cell *β*. Larger values of its coefficient Λ_*αβ*_ generate stronger tension or weaker cell-cell adhesion. Λ_*αβ*_ is a key parameter to regulate tissue fluidity, that is, how the tissue behaves like liquid when external forces are applied. For smaller values for Λ_*αβ*_, the effect of area constraint is relatively large and each cell moves so as to maintain its apical area as near to the natural value as possible. In addition, a larger cell-cell contact length is preferable (i.e., the energy level is lowered). This leads to easier cell deformation and more frequent cell-cell rearrangements when forces (due to division of surrounding cells) act on the cell. In contrast, when Λ_*αβ*_ takes larger values, the force of isotropic shrinking shows a relative increase and a shorter cell-cell contact length is preferable, making cellular deformation and cell-cell rearrangement more difficult. Consequently, the tissue fluidity decreases. The third term of Eq (20) is the elastic energy of the cell perimeter *L*_*α*_. Its coefficient Γ_*α*_ determines the magnitude of apical contractility. In the first three (2nd-4th) subsections of the Results, we considered the pure population case where all cells have the same mechanical properties. Thus Λ_*αβ*_ and Γ_*α*_ are simply referred to as Λ and Γ. In the last two subsections of the Results, it is possible that the coefficient of line tension Λ can vary between cells. The effective value of Λ at the contact surface between cells that have different values (Λ_*α*_ and Λ_*β*_) might depend on underlying molecular mechanisms. For example, when the coefficient of line tension is regulated by the amount of homophilic adhesive molecules, its effective value Λ_*αβ*_ on the focal edge is regarded as max(Λ_*α*_, Λ_*β*_) or simply approximated by the average (Λ_*α*_+Λ_*β*_)/2 (Eq (21)). Although the results obtained in this study did not change qualitatively, we chose to use the former, i.e., max(Λ_*α*_, Λ_*β*_), for the simulations performed in the final subsection of the Results.

The model is usually used after non-dimensionalization that is done by introducing the following variables and parameters:

$$\begin{array}{l} \hat{t}\equiv \delta t,\;{\hat{r}}_{i}\equiv \frac{r_{i}}{\sqrt{A_{0}}},\;\hat{U}\equiv \frac{U}{ΚA_{0}{}^{2}},\;\hat{\eta }\equiv \frac{\eta \delta }{ΚA_{0}},\\ {\hat{\Lambda }}_{\alpha \beta }\equiv \frac{\Lambda_{\alpha \beta }}{ΚA_{0}},\;{\hat{\Gamma }}_{\alpha }\equiv \frac{\Gamma_{\alpha }}{ΚA_{0}},\;{\hat{l}}_{\alpha \beta }\equiv \frac{l_{\alpha \beta }}{\sqrt{A_{0}}},\;{\hat{L}}_{\alpha }\equiv \frac{L_{\alpha }}{\sqrt{A_{0}}}. \end{array}$$

Then Eqs (19)–(21) are represented as:

$$\hat{\eta }\frac{d{\hat{r}}_{i}}{d\hat{t}}=-\frac{\partial \hat{U}}{\partial {\hat{r}}_{i}},$$ (22)

$$\hat{U}=\frac{1}{2}\sum_{\alpha }^{}{\left({\hat{A}}_{\alpha }-1\right)}^{2}+\sum_{<\alpha ,\beta >}^{}{\hat{\Lambda }}_{\alpha \beta }{\hat{l}}_{\alpha \beta }+\sum_{\alpha }^{}\frac{{\hat{\Gamma }}_{\alpha }}{2}{\hat{L}}_{\alpha }{}^{2},$$ (23)

$${\hat{\Lambda }}_{\alpha \beta }=\{\begin{array}{c} \text{ max}({\hat{\Lambda }}_{\alpha },{\hat{\Lambda }}_{\beta })\\ \;({\hat{\Lambda }}_{\alpha }+{\hat{\Lambda }}_{\beta })/2 \end{array},$$ (24)

where the hat symbol “^” indicates a dimensionless quantity. $\hat{\Lambda }$ and $\hat{\Gamma }$ are the non-dimensionalized free parameters representing the weights relative to area constraint of the line tension/adhesion and the apical contractility elasticity, respectively. We used ($\hat{\Lambda }$, $\hat{\Gamma }$) = (0.14, 0.04) as a reference parameter set. For simplicity, we omitted the hat symbol from the normalized quantities in every part except here.

Regarding cell division, we introduced a clock representing the cell cycle for each cell. The clock goes forward linearly with time. When the timer reaches a threshold *T*, a cell enters the mitotic phase of division. To prevent the synchronization of cell division, we included 20% randomness (uniformly-distributed) into the cell cycle time. During the mitotic phase, the natural area *A*_0_ in Eq (20) increases linearly with time, and the actual area of the cell (*A*_*α*_ in Eq (20)) expands to double the area just before entering the mitotic phase. When the area doubles, the cell divides with an axis through its center, then the cell cycle clock is reset to zero and a new cycle time *T* is chosen. We examined another method for cell division in which a cell does not have a mitotic phase, and the cell divided immediately when the clock reached the threshold described above. In this case, the simulation results were qualitatively the same as those in the case with the mitotic phase. This is consistent with the previous study [56]. Regarding the division orientation, we modeled it as a random variable obeying von Mises distribution *f*(*θ*;*κ*) around the shortest axis with a parameter *κ* controlling its variation (Fig 2B). The shortest axis was obtained from the elliptical approximation of a cell using its vertices (i.e., tricellular junctions). This modeling of division orientation was because of Hertwig’s rule [64]. Recently, Bosveld et al. [65] examined the mechanism of Hertwig’s rule by observing spatial distribution of tricellular junctions.

As a consequence of push-pull dynamics between cells in a growing tissue, cells rearrange spatially and cell elimination occurs. The rearrangement is implemented by edge reconnection as shown in Fig 2C. This process is called a T1-process in the physics of foam [66] and occurs when the edge length is less than T1-threshold *θ*_T1_. The elimination is implemented simply by removing a cell whose area is less than T2-threshold *θ*_T2_, which is called a T2-process. We used *θ*_T2_ = 0.2 the value of which reflects experimental observations [4]. We also confirmed that our results did not qualitatively change when we used different values for *θ*_T2_ (specifically, *θ*_T2_ = 0.05, 0,1, 0.3). As shown in the 4th subsection of the Results, cell size is highly correlated with its stress magnitude defined below, and thus our assumption of the criterion for MCE, i.e. the existence of a cell size threshold for MCE, also includes another criterion, the existence of a threshold for stress magnitude acting on a cell.

All growth simulations started with 250 cells. Edges of the boundary have stronger line tension (3Λ_*α*_) than inner edges for maintaining the circular shape of the entire tissue and preventing occasional complicated boundary shapes such as protrusions, etc. In calculating the statistics, we excluded the boundary cells, and we also confirmed that the line tension imposed at the boundary we used only minimally affected our results, especially pertaining to the cell elimination rate.

### Two types of stress tensors as discrete representations of Cauchy’s stress

In this study, the stress within a tissue was evaluated by two types of stress tensors used in recent papers [46,47]. These tensors are discrete representations of Cauchy’s stress and are defined by using the forces acting on vertices that compose each polygonal cell. Our analysis is based on the assumption that cell deformation occurs at a sufficiently slow speed that viscous forces are negligible and cells can be considered quasi-static (the forces acting on each vertex were considered approximate to the mechanical equilibrium). In actuality, in our simulations each cell or vertex only moves in response to cell division, and the cell cycle time (for the reference case) is long enough compared to the relaxation time of the vertex dynamics model (except for cells around the division point, the movement of almost all cells is very slow). To date, several methods have been proposed for calculating the force/stress tensor in an epithelial cell population. For example, Chiou et al. and Brodland et al. have discussed in detail how to address stress within a tissue [58,59].

The force acting on vertex *i* of cell *α* is composed of pressure inside the cell, *P*_*α*_ and tensions on the two edges that are linked to the vertex, *T*_*ij*,*α*_, *T*_*ik*,*α*_. The edge tension involving the cell *α* was assumed to be half of the tension totally acting on the focal edge, *T*_*ij*_, and the remaining half was allotted to the other cell *β* that shares the edge, i.e., *T*_*ij*_ = *T*_*ij*,*α*_+*T*_*ij*,*β*_ = 2*T*_*ij*,*α*_ (Fig 6A).

In one stress tensor (denoted by ***σ***^(*A*)^_*α*_) [46], the force acting at each point along an edge was calculated by linear interpolation using the force vectors on the two vertices at the ends of the edge **F**_*i*_ and **F**_*j*_:

$$\sigma^{\left(A\right)}{}_{\alpha }=\frac{1}{A_{\alpha }}\text{sym}\left[\oint_{c}F⊗r\right]≅\frac{1}{A_{\alpha }}\text{sym}\left[\sum_{<i,j>}\int_{0}^{1}F_{ij}(\lambda )⊗r_{ij}(\lambda )\;d\lambda \right],$$ (25)

where **F**_*ij*_(*λ*) = *λ* **F**_*i*_+(1-*λ*)**F**_*j*_ and **r**_*ij*_(*λ*) = *λ* **r**_*i*_+(1-*λ*)**r**_*j*_. **r**_*i*_ and **r**_*j*_ are the positional vectors of vertices *i* and *j* from the cell center. The force vector acting on each vertex is defined as:

$$F_{i}=P_{\alpha }n_{jk}+T_{ij,\alpha }\frac{r_{ij}}{\left\|r_{ij}\right\|}+T_{ik,\alpha }\frac{r_{ik}}{\left\|r_{ik}\right\|},$$ (26)

where **n**_*jk*_ is the normal vector to the segment connecting the adjacent vertices of the focal vertex (see also Fig 6A), and **r**_*ij*_ = **r**_*j*_−**r**_*i*_, **r**_*ik*_ = **r**_*k*_−**r**_*i*_. For details, see Alim et al. [46].

The other stress tensor (denoted by ***σ***^(*B*)^_*α*_) was calculated using the pressure *P*_*α*_, identity matrix **I**, tension *T*_*ij*,*α*_ and positional vector **r**_*ij*_ from the focal vertex *i* to the adjacent one *j* [47]:

$$\sigma^{\left(B\right)}{}_{\alpha }=-P_{\alpha }I+\sum_{<i,j>}\frac{T_{ij}}{A_{\alpha }}\frac{r_{ij}⊗r_{ij}}{\left\|r_{ij}\right\|}.$$ (27)

For both stress tensors ***σ***^(*A*)^_*α*_ and ***σ***^(*B*)^_*α*_, *P*_*α*_ and *T*_*ij*,*α*_ were calculated as follows:

$$P_{\alpha }=-\frac{\partial U}{\partial A_{\alpha }}=-(A_{\alpha }-1),$$ (28)

$$T_{ij,\alpha }=\frac{\partial U}{\partial l_{ij}}=\frac{\Lambda_{\alpha \beta }}{2}+\Gamma_{\alpha }L_{\alpha }.$$ (29)
